# Children With Dyslexia and Familial Risk for Dyslexia Present Atypical Development of the Neuronal Phonological Network

**DOI:** 10.3389/fnins.2019.01287

**Published:** 2019-11-29

**Authors:** Magdalena Łuniewska, Katarzyna Chyl, Agnieszka Dębska, Anna Banaszkiewicz, Agata Żelechowska, Artur Marchewka, Anna Grabowska, Katarzyna Jednoróg

**Affiliations:** ^1^Laboratory of Language Neurobiology, Nencki Institute of Experimental Biology, Polish Academy of Sciences, Warsaw, Poland; ^2^Laboratory of Brain Imaging, Neurobiology Center, Nencki Institute of Experimental Biology, Polish Academy of Sciences, Warsaw, Poland; ^3^RITMO Centre for Interdisciplinary Studies in Rhythm, Time and Motion, University of Oslo, Oslo, Norway; ^4^Faculty of Psychology, SWPS University of Social Sciences and Humanities, Warsaw, Poland

**Keywords:** dyslexia, familial risk for dyslexia, phonological processing, phonological development, fMRI, longitudinal design

## Abstract

Learning to read changes the brain language system. Phonological processing is the language domain most crucial for reading, but it is still unknown how reading acquisition modifies the neural phonological network in children who either develop dyslexia or are at risk of dyslexia. For the two first years of formal education, we followed 90 beginning readers with (*n* = 55) and without (*n* = 35) familial risk of dyslexia who became typical readers (*n* = 70) or developed dyslexia (*n* = 20). We used functional magnetic resonance imaging to identify the neural correlates of phonological awareness using an auditory rhyme judgment task. This task was applied when participants were starting formal education, and repeated 2 years later. By applying two alternative group splits, we analyzed the effects of dyslexia and the effects of familial risk of dyslexia separately. We found that the phonological brain network undergoes reorganization during the first 2 years of formal education. This process proceeds differently depending on the presence of a familial history of dyslexia and reading impairment. Typical readers without risk for dyslexia activate structures responsible for phonological processing already at the beginning of literacy. This group shows reduced brain activation over time during phonological processing, perhaps due to automatization of phonological skills. Children who develop reading impairment present a delay in the development of phonological structures such as the bilateral superior temporal gyri, left middle temporal gyrus, right insula and right frontal cortex, where we observed time and group interaction. Finally, typical readers with familial risk of dyslexia also present an atypical development of the neural phonological structures, visible both at the beginning of reading instruction and 2 years later. These children used a presumably efficient neural mechanism of phonological processing, based on the activation of the precentral and postcentral gyri, and achieved a typical level of phonological awareness.

## Introduction

Learning to read is a long-lasting process which requires mastering a number of skills. Phonological skills, i.e., understanding that spoken words are composed of discrete sounds which can be mapped onto letters, are particularly important, and a deficit in phonological skills is a common etiology of developmental dyslexia (Wagner and Torgesen, 1987; Ziegler et al., 2010). The brain network responsible for phonological processing includes mostly left hemisphere areas like the inferior parietal lobule (including supramarginal and angular gyri), inferior frontal cortex, postcentral and precentral gyri, superior and middle temporal gyri, and fusiform and dorsolateral prefrontal cortex (Poldrack et al., 1999; Booth et al., 2006; Hoeft et al., 2006; Bitan et al., 2007; Cao et al., 2008; Kovelman et al., 2012). Patterns of brain activation during phonological processing differ between dyslexic and typical readers and have been observed with functional magnetic resonance imaging (fMRI; Richlan et al., 2011). However, only a few fMRI experiments have explored phonological processing independent of reading abilities using a task based on spoken language (Desroches et al., 2010; Kovelman et al., 2012; Raschle et al., 2012, 2014; Dębska et al., 2016; Yu et al., 2018). Studies on dyslexic and typically reading children have produced inconsistent results: Desroches et al. (2010) reported hypoactivation of the left fusiform gyrus in the dyslexic group while Kovelman et al. (2012) found hypoactivation only in the left dorsolateral prefrontal cortex.

In order to explore patterns of brain activation independent of reading experience and already present before schooling, Raschle et al. (2014) recruited pre-reading children with and without a familial history of dyslexia. Children with familial history of dyslexia constitute a common risk group and tend to have around a 45% chance to develop a reading disorder (Snowling and Melby-Lervåg, 2016). Children with a familial history of dyslexia exhibited underactivation during phonological processing in the bilateral occipito-temporal and left temporoparietal areas, as well as in the cerebellum (Raschle et al., 2014). They also showed disruption in the left frontal brain regions during auditory processing (Raschle et al., 2014). Dębska et al. (2016), who tested Polish-speaking pre-schoolers and first-graders, reported similar results. Children with a familial history of dyslexia showed reduced activation of many brain regions, including the left occipito-temporal cortex. For this particular region, reduced activation was observed only among pre-schoolers in the familial risk group. At the same time, a grade effect of lower activity in first-graders compared to kindergarten pupils was observed exclusively in children without familial history of dyslexia. This cross-sectional study raises the question as to what the connection is between familial history of dyslexia and formal reading instruction. It may be that the phonological network engaged by pre-schoolers is not essential for phonological processing during the time when both phonological awareness and reading are becoming more fluent. As a result, any differences between children with and without familial risk may only be visible at the pre-reading state. However, it is still not clear how development and literacy acquisition affect differences in phonological processing between dyslexic and typically reading children, and whether familial risk for dyslexia plays a role in this process.

Literacy affects the brain language network (Dehaene et al., 2015) since reading acquisition supports the development of more advanced phonemic skills through experience with an alphabet (Bentin, 1992). Phonological processing is also affected by learning to read. The level of reading skills at the beginning of schooling is a predictor of growth in phonological awareness in the following year (Nation and Hulme, 2011). Reading skills support the development of subvocal rehearsal which, in turn, improves performance in verbal working memory tasks (Demoulin and Kolinsky, 2016). However, it is still unknown how reading development affects neural correlates of auditory phonological processing, even though phonology is a language domain crucial for reading. Two cross-sectional studies provided evidence for age-related increases of brain activation in the phonological processing network in alphabetic languages (Cone et al., 2008; Brennan et al., 2013). Specifically, English-speaking typical readers during rhyme judgment showed increased activation with age in the left dorsal inferior frontal and temporal gyri (Cone et al., 2008; Brennan et al., 2013) and inferior parietal cortex (Brennan et al., 2013). However, in cross-sectional studies, age-related effects may also come from existing between-group differences independent of age or reading experience. So far only one study has used a longitudinal design to explore the development of the phonological network in typical readers from the beginning of formal education until the emergent reading stage (5–7–9 years of age, Yu et al., 2018). They found a developmental decrease in activation in the left inferior parietal cortex and bilateral precuneus. This decrease suggests that experience in reading induced specialization of brain regions responsible for phonological processing. As participants were restricted to typical readers without familial risk of dyslexia, it is unknown how the phonological network develops in atypical reading development where children have a familial history of dyslexia or reading disabilities.

Here, we aim to longitudinally explore changes in the phonological processing network that occur when children with and without familial risk for dyslexia learn to read, and to follow these changes as they either become proficient readers or develop dyslexia. First, we explore the development of the neural basis of phonological processing in children who acquire reading typically and children who develop dyslexia during the first 2 years of their school education. We hypothesize that typical readers will show reduced brain activity in the language processing network in later stages of reading development as compared with the early stage, in-line with experience-induced fine-tuning reported previously (Yu et al., 2018). We expect children with dyslexia to display behavioral and brain activation differences as compared to typical readers at both early and later stages of reading development. In particular, we expect children with dyslexia to present low accuracy in both reading and phonological assessments at all measurement points and to show hypoactivation of left hemisphere structures (as in Desroches et al., 2010; Kovelman et al., 2012).

Second, by applying alternative group splitting, we examine the impact of familial history of dyslexia on the neural correlates of phonological processing during the same time range of the first years of education. We expect that the phonological network of children with familial risk for dyslexia who become typical readers should differ from that of typical readers without familial history of dyslexia at the beginning of literacy acquisition. Those children with a family history of dyslexia that become typical readers should show a vast hypoactivation of the typical phonological network (as in Raschle et al., 2014; Dębska et al., 2016). We hypothesize that a familial history of dyslexia has a persistent impact on brain function, even in typically reading children. Therefore, we expect to find the effects of familial risk not only at the beginning of education, but also in later stages of reading development. Presumably, the effects of familial history of dyslexia will be observed independent of dyslexia itself.

## Materials and Methods

### Participants

All participants took part in a longitudinal study approved by the Warsaw University Ethical Committee. The study consisted of three time points, a year apart from each other. The first and the third time points involved both behavioral and fMRI sessions. The second time point was limited to behavioral testing.

At the first time point, 120 native Polish-speaking children were recruited from first grade and kindergarten classes. Due to the educational reform taking place in Poland during our study, children could begin formal school education at age six or seven, depending on their parents’ choice. Therefore, the age range of children who attended first grade in September was 5.9 (if a child was born in December and her/his parents decided that she/he would begin schooling as 6-year-old) to 7.8 (if a child was born in January and her/his parents decided that she/he would begin schooling as 7-year-old). This massive variation of age of first-graders is reflected in our sample. Similarly, there was a variation in age among children who attended the last year of kindergarten, who were on average 6 months younger than the first-graders in our sample. Because of the relatively small difference in age between the two groups, we decided to recruit children from both the first grade and kindergarten. Though formal reading training was supposed to start in elementary school, children were already taught letters in kindergarten.

The participants had typical or high IQs (higher than 25. percentile as measured with Raven Matrices), were monolingual, right-handed (as reported by parents and confirmed by experimenters during testing, when children were supposed to use the hand they prefer for writing), and born at term (≥37 weeks of pregnancy). None of them had any history of neurological illness or brain damage and no prior symptoms of ADHD as reported by their parents. From the group of 102 participants in our previous study (Dębska et al., 2016), nine children either left the study due to moving to another city or losing interest in the project. Additionally, data from three children (the oldest one and the two youngest ones) were excluded from analyses in order to clearly separate the age range of participants during the first and the third time points.

The current study included 90 children (53 girls and 37 boys) aged 5.94–7.95 years (6.91 years on average) at the first time point, 6.99–9.43 (7.88 years on average) at the second time point, and 8.05–10.05 years (8.95 years on average) at the third time point. At the first time point, 27 participants were attending the last year of kindergarten and 63 were first graders. At consecutive time points, children progressed in their education until, at the third time point, there were 27 second graders and 63 third graders. The distribution of children who attended school and kindergarten did not differ between children with a familial history of dyslexia and without. There was also no difference in distribution between children diagnosed later with dyslexia and typical readers. Therefore, the results were affected by the school grade to the same extent in all subgroups.

There were 55 children with familial history of dyslexia and 35 children without familial history of dyslexia. Children with a familial history of dyslexia were identified as those who had a first-degree relative with a formal diagnosis of developmental dyslexia or at least one parent who scored more than 0.4 points in the Adult Reading History Questionnaire (ARHQ, Lefly and Pennington, 2000), as specified in Black et al. (2012). These criteria overlapped in 32 children while 23 children fulfilled only the questionnaire criterion. There were no children who had a first-degree relative with a diagnosis of dyslexia and for which both parents scored below 0.4 points in the questionnaire. The two groups of children whose parents scored high in ARHQ and had a first-degree relative with diagnosis of dyslexia (maternal ARHQ: *M* = 0.35, *SD* = 0.15, paternal ARHQ: *M* = 0.45, *SD* = 0.15) and children whose parents scored high in ARHQ but had no formal diagnosis of dyslexia in family (maternal ARHQ: *M* = 0.42, *SD* = 0.12, paternal ARHQ: *M* = 0.38, *SD* = 0.16) did not differ in maternal or paternal ARHQ average scores (maternal: *t*(53) = 1.80, *p* = 0.078, 95% CI DV = [−0.01; 0.14], *d* = 0.50; paternal: *t*(48) = 1.68, *p* = 0.100, 95% CI DV = [−0.16; 0.015], *d* = 0.48). We used the questionnaire measure due to the fact that in Poland, reading impairment was not diagnosed when the parents of our participants were school aged. However, according to the norming study of the Polish version of ARHQ, the criterion of scoring more than 0.4 points is fulfilled by 11% of mothers and 16% of fathers (Krasowicz-Kupis et al., 2014).

The third time point included a formal diagnosis of dyslexia and made it possible to select a group of children with dyslexia (*n* = 20). Children selected as having a reading disorder achieved low scores (at least 1 SD below the population mean, which corresponds to below 16 percentile) in at least two reading subtests (out of four: single-word reading, pseudo-word reading, reading with lexical decision, and text reading). Children who achieved a low score in no more than one reading subtest were assigned to the typically reading group. Children assessed as dyslexic achieved low scores in 3.05 tests (out of four) on average (*SD* = 0.69), and children assessed as typically reading scored low in 0.23 tests (out of four) on average (*SD* = 0.43). The dyslexic group included 15 children with a familial history of dyslexia and 5 children without a familial history of dyslexia, whereas the group of typical readers (*n* = 70) included 40 children with a familial history of dyslexia and 30 children without such risk factor.

Children with dyslexia did not differ from typical readers in terms of age at each time point, sex, grade, or verbal and non-verbal IQ (as measured with WISC-R). Although their parental socioeconomic status, paternal score in the Adult Reading History Questionnaire, and performance IQ (measured with Raven Matrices) was slightly lower than in the typically reading group, these differences did not survive Bonferroni correction for multiple comparisons (see Table 1). Similar effects were observed when the groups were restricted to children with a familial history of dyslexia (see Supplementary Table S1).

**TABLE 1:** Participants’ characteristics: whole sample (TR, typical readers; DR, children with dyslexia; FHD−, children without a familial history of dyslexia; FHD+, children with a familial history of dyslexia; TP, time point; ARHQ, Adult Reading History Questionnaire).

	**TR**	**DR**		**FHD−**	**FHD+**	
	***n* = 70**	***n* = 20**		***n* = 35**	***n* = 55**	
Gender (boys/girls)	26/44	11/9	χ^2^ = 2.05; *p* = 0.152	13/22	24/31	χ^2^ = 0.37; *p* = 0.542
Grade at TP1 (kindergarten/first grade)	18/52	9/11	χ^2^ = 2.76; *p* = 0.097	12/23	15/40	χ^2^ = 0.50; *p* = 0.479
Age at TP1 and TP3 (years)	6.94 (0.49) 8.98 (0.50)	6.81 (0.46) 8.85 (0.46)	*F*(1,88) = 0.94; *p* = 0.336 η_*p*_^2^ = 0.011	6.87 (0.49) 8.92 (0.52)	6.94 (0.49) 8.96 (0.48)	*F*(1,88) = 0.42; *p* = 0.519 η_*p*_^2^ = 0.005
Socioeconomic status	50.00 (9.97)	41.73 (13.35)	*U* = 457; *p* = 0.018 *d* = 0.78	50.81 (8.09)	46.48 (12.68)	*U* = 801; *p* = 0.181; *d* = 0.39
ARHQ mother	31.10 (13.63)	34.25 (16.92)	*U* = 650; *p* = 0.627 *d* = 0.22	21.97 (8.01)	38.05 (14.08)	*U* = 324; *p* < 0.001^∗^ *d* = 1.34
ARHQ father	33.83 (14.81)	42.06 (15.81)	*U* = 383; *p* = 0.052 d = 0.56	25.25 (7.27)	42.12 (15.49)	*U* = 270; *p* < 0.001^∗^ *d* = 1.32
Home literacy	36.34 (5.98)	32.89 (5.69)	*U* = 409; *p* = 0.014 *d* = 0.59	37.59 (5.77)	34.30 (5.94)	*U* = 569; *p* = 0.004^∗^ *d* = 0.57
Number of letters known at TP1 and TP2	51.36 (15.07) 63.04 (2.57)	30.50 (19.53) 58.90 (8.84)	*F*(1,88) = 28.37, *p* < 0.001^∗^ η_*p*_^2^ = 0.244	46.97 (17.68) 61.97 (5.42)	46.56 (18.78) 62.22 (4.73)	*F*(1,88) = 0.00, *p* = 0.972 η_*p*_^2^ = 0.000
Raven Matrices IQ (sten)	7.70 (1.16)	6.95 (1.64)	*U* = 505; *p* = 0.050 *d* = 0.59	7.71 (1.10)	7.42 (1.42)	*U* = 876; *p* = 0.458 *d* = 0.22
WISC-R IQ	123.52 (12.51)	117.25 (14.09)	*U* = 519; *p* = 0.093 *d* = 0.49	124.15 (13.36)	120.85 (12.84)	*U* = 796; *p* = 0.238 *d* = 0.26
Digit span (number of repeated strings)	6.59 (1.77)	5.95 (1.50)	*U* = 508; *p* = 0.054 *d* = 0.38	6.69 (1.62)	6.29 (1.79)	*U* = 797; *p* = 0.157 *d* = 0.23
Syllable span (number of repeated strings)	8.73 (2.59)	7.85 (2.62)	*U* = 547; *p* = 0.134 *d* = 0.34	8.91 (2.42)	8.29 (2.71)	*U* = 835; *p* = 0.285 *d* = 0.24
Vocabulary at TP1 (percentile)	76.11 (21.98)	57.71 (24.43)	*U* = 166; *p* = 0.010 *d* = 0.83	73.7 (21.91)	70.34 (25.09)	*U* = 372; *p* = 0.627 *d* = 0.14
Word reading at TP1, TP2, and TP3 (items read/minute)	19.60 (18.70) 50.39 (22.72) 74.17 (23.69)	3.80 (5.22) 20.60 (9.77) 35.3 (8.67)	*F*(1,87) = 40.86, *p* < 0.001^∗^ η_*p*_^2^ = 0.320	17.2 (20.38) 44.31 (24.36) 69.15 (29.76)	15.38 (16.28) 43.42 (23.98) 63.15 (24.73)	*F*(1,87) = 0.50, *p* = 0.483 η_*p*_^2^ = 0.006
Pseudo-word reading at TP1, TP2, and TP3 (items read/minute)	15.8 (13.41) 33.34 (10.20) 42.00 (11.21)	3.40 (4.98) 17.25 (7.96) 26.70 (6.66)	*F*(1,86) = 40.77, *p* < 0.001^∗^ η_*p*_^2^ = 0.322	13.53 (13.16) 30.49 (12.27) 39.82 (13.56)	12.69 (13.15) 29.31 (11.60) 37.78 (11.28)	*F*(1,86) = 0.44, *p* = 0.509 η_*p*_^2^ = 0.005
Phoneme analysis TP1, TP2, and TP3 (items solved)	7.79 (4.00) 10.26 (2.58) 10.61 (2.63)	3.05 (3.17) 8.65 (3.80) 10.30 (2.49)	*F*(1,87) = 12.49, *p* < 0.001^∗^ η_*p*_^2^ = 0.126	7.29 (4.30) 10.29 (2.28) 10.91 (2.14)	6.38 (4.30) 9.65 (3.30) 10.31 (2.83)	*F*(1,87) = 1.69, *p* = 0.198 η_*p*_^2^ = 0.019
Phoneme deletion TP1, TP2, and TP3 (items solved)	4.84 (4.36) 10.09 (3.44) 13.55 (4.60)	1.30 (2.36) 5.10 (4.47) 8.50 (4.81)	*F*(1,87) = 25.46, *p* < 0.001^∗^ η_*p*_^2^ = 0.226	4.06 (4.56) 8.91 (3.67) 12.76 (4.66)	4.05 (4.10) 9.02 (4.56) 12.20 (5.36)	*F*(1,87) = 1.69, *p* = 0.198 η_*p*_^2^ = 0.019
Rapid naming colors and objects TP1, TP2, and TP3 (seconds)	126.16 (27.70) 107.65 (20.44) 93.97 (16.50)	153.50 (42.22) 129.50 (34.26) 116.35 (25.53)	*F*(1,87) = 17.73, *p* < 0.001^∗^ η_*p*_^2^ = 0.169	138.32 (36.57) 112.12 (21.66) 98.76 (21.75)	128.58 (30.89) 112.84 (28.10) 99.15 (20.67)	*F*(1,87) = 0.04, *p* = 0.852 η_*p*_^2^ = 0.000
Rapid naming letters and digits TP2 and TP3 (seconds)	61.91 (13.71) 51.66 (9.30)	86.47 (27.33) 66.00 (11.97)	*F*(1,83) = 33.41, *p* < 0.001^∗^ η_*p*_^2^ = 0.287	65.85 (21.35) 54.15 (12.28)	67.44 (18.84) 54.77 (10.89)	*F*(1,83) = 0.11, *p* = 0.749 η_*p*_^2^ = 0.001

*^∗^Remains significant after Bonferroni correction for multiple comparisons, i.e., six to ten comparisons at each TP. For ANOVA analyses, values of *F, p*, and η_*p*_^2^ are reported for group effects.*

Groups of children with and without familial history of dyslexia did not differ in age at each time point, sex, grade, parental socioeconomic status, or IQ. Children with a familial history of dyslexia had significantly higher maternal and paternal scores in the Adult Reading History Questionnaire than children without familial risk (see Table 1), as it was the group division criterion. Similar effects were observed when the groups were restricted to typically reading children (see Supplementary Table S1).

### Experimental Design: Behavioral Measures

Participants completed three phases of behavioral tests which included measuring reading skills, letter knowledge, rapid naming, and phonological awareness (all time points), and language and cognitive skills (the first time point). Standard tests designed for dyslexia diagnosis were applied at the third time point. Parents of participants completed questionnaires about their reading history (ARHQ, Lefly and Pennington, 2000), as well as about their home literacy environment.

Letter knowledge, word and pseudo-word reading, elision and phoneme analysis were measured at each time point with the same test battery (Szczerbiński and Pelc-Pêkala, 2013). Rapid automatized naming was tested with subtests of object and color naming at the first time point, and object, color, letter, and digit naming at further time points (Fecenec et al., 2013). Language skills were assessed with the Picture Vocabulary Test: Comprehension (vocabulary assessment at the first time point; Haman et al., 2012), and selected subtests of Test of Language Development (vocabulary and grammar assessment at the second time point; Smoczyńska et al., 2015). Intelligence was assessed with Raven’s Colored Progressive Matrices (Szustrowa and Jaworowska, 2003) at the first time point, and with Wechsler Intelligence Scale for Children – Revised (WISC-R; Matczak et al., 2008) at the second time point. Syllable and digit span were measured at the first time point with tasks in which series of increasing length were repeated by participants, and the total number of correctly repeated series was used as the outcome measure.

At the third time point, a standardized battery of tests for dyslexia diagnosis was applied (Bogdanowicz et al., 2009). The battery consisted of nine tests: four of them assessing reading, two assessing writing, and three measuring phonological skills. Reading tests included word reading, pseudoword reading, reading with lexical decision, and text reading with comprehension control. Writing was measured with word writing and text writing. Phonological abilities were assessed using phoneme deletion, pseudoword repetition, and a battery of tasks (phoneme analysis and synthesis, syllable analysis and synthesis) based on pseudowords.

### Statistical Analyses

We divided the analyses into two parts: (A) concerning the effects of dyslexia and (B) concerning the effects of familial history of dyslexia. For each time point we compared performance in behavioral tests between children who developed typical reading skills and children with dyslexia for the whole sample. Next, we repeated the analyses for the sample restricted to children with familial history of dyslexia to further explore if differences between typical and dyslexic readers were driven by reading ability regardless of familial risk. Similarly, all comparisons were performed between children with and without familial history of dyslexia for the whole sample and then repeated for only typical readers. For comparisons between groups of unequal sizes (typical readers vs. children with dyslexia in the whole sample and in children with familial history of dyslexia; children with familial history of dyslexia vs. children without familial history of dyslexia) non-parametric methods were applied, whereas for comparison of typical readers with and without familial history of dyslexia, parametric tests were used. Each series of comparisons was followed by Bonferroni corrections.

The optimal way to compare the effects of presence of reading impairment and familial risk of dyslexia in one model, would be to run 2 × 2 comparisons. However, the small size of the group of children without familial risk of dyslexia, who developed reading impairment (*n* = 5) did not allow us to run this type of analyses. Therefore, we applied the same model of analyses as used before for research of white matter volume in a similar group of children with and without familial risk of dyslexia and with and without reading impairment (Vanderauwera et al., 2017). This involved analyzing the effects of dyslexia first in whole sample, then in children with familial history of dyslexia, and the effects of familial history of dyslexia first in whole sample, then in typical readers.

### Experimental Design: fMRI Tasks

The same fMRI tasks were applied at the first and the third time points and have been described by Dębska et al. (2016). Twenty noun pairs were presented to both ears of participants via headphones. The noun pairs were accompanied by pictures depicting the words. Subjects viewed the stimuli on a back-projection screen through an angled mirror. The exact list of stimuli is provided as Supplementary Table S2. After each pair, children had to decide whether the words rhymed or not (Rhyme task), similar to Kovelman et al. (2012). The control experiment included exactly the same stimuli, but the participants’ task was to assess whether the words were spoken by speakers of the same gender or not (Voice task), similar to Raschle et al. (2012). The yes/no decision was made by pressing the corresponding button. Both tasks were contrasted with a rest condition. During the rest condition, children looked at a fixation cross for the duration of the block. The accuracy and reaction times were analyzed at both TPs using repeated-measures ANOVA.

The procedure was the same for both time points. Children were familiarized with the task in a mock-scanner using items not included in the following scanning session. The experimental scheme consisted of two functional runs: one with the experimental Rhyme task and one with the control Voice task. The timing and duration of tasks were identical. The order of runs was counterbalanced between the children and reversed at the third time point (compared to the first time point). Each word was presented to children via headphones, and at the same time the picture of its meaning appeared on the screen for 2 s. After that, the second word was played and the second picture appeared for 2 s. Then, a question mark appeared for 2 s prompting the child to give an answer. Each run consisted of ten blocks: five blocks with stimulation, with four trials per block, and five with the rest condition, each lasting 24 s. Half of the trials matched regarding rhyme and half of the words were spoken by the same gender voice. Stimuli were presented using Presentation software (Neurobehavioral Systems).

## Results

### Effects of Dyslexia

#### Behavioral Results

**FIGURE 1:**
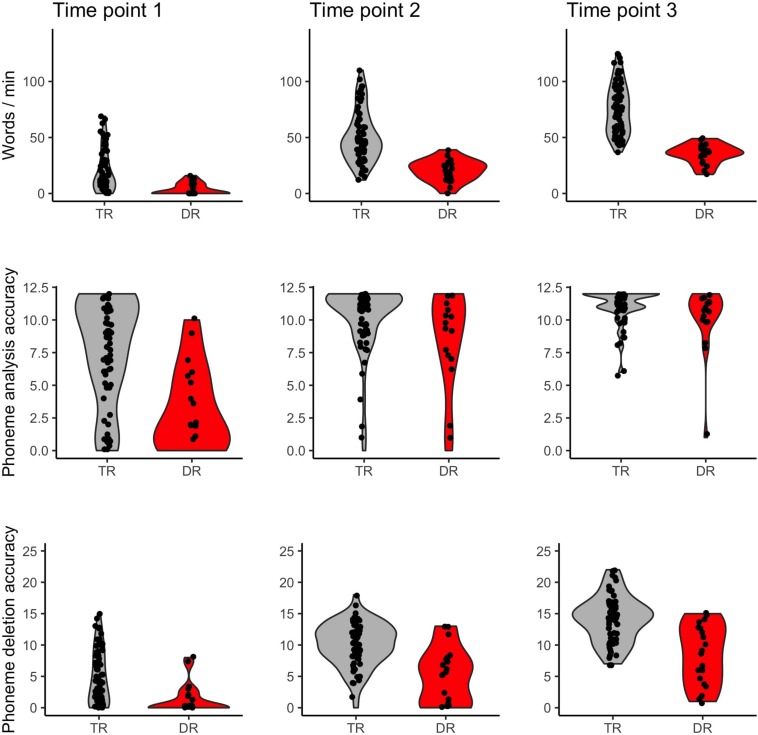
Word reading, phoneme analysis, and phoneme deletion of typical readers (TR) and children with dyslexia (DR) across three time points.

Children with dyslexia had lower performance than typical readers in early reading and reading related tests, already at the first time point when children had only just started formal education (see Table 1 and Figure 1). Differences between these two groups were observed in letter knowledge (the first two time points), word and pseudoword reading (at each time point), phoneme analysis (only at the first time point due to ceiling effect at later time points), phoneme deletion (at each time point), rapid automatized naming of colors and objects (the first and the third time points), and rapid automatized naming of letters and digits (the second and the third time points). However, vocabulary and working memory performance measured with digit and syllable span of children with dyslexia did not differ from the typically reading group at the first time point. Additionally, in all tests from the normalized battery for dyslexia diagnosis, children with dyslexia scored significantly lower than typically reading children (see Table 2). This pattern of results was not qualitatively different when the group was restricted to children with a familial history of dyslexia (see Supplementary Table S1 for demographic and reading related tests and Supplementary Table S3 for dyslexia diagnosis tests).

**TABLE 2:** Tests diagnosing dyslexia: whole sample (TR, typical readers; DR, children with dyslexia; FHD−, children without a familial history of dyslexia; FHD+, children with a familial history of dyslexia).

	**TR**	**DR**		**FHD−**	**FHD +**	
	***n* = 70**	***n* = 20**		***n* = 55**	***n* = 35**	
Word reading	6.55 (1.80) 0.53 (0.90)	3.55 (1.43) −0.98 (0.72)	*U* = 120; *p* < 0.001^∗^ *d* = 1.76	6.03 (2.05) 0.27 (1.03)	5.78 (2.19) 0.14 (1.10)	*U* = 907; *p* = 0.807; *d* = 0.12
Pseudo-word reading	5.62 (1.45) 0.06 (0.72)	2.90 (1.33) −1.30 (0.67)	*U* = 123; *p* < 0.001^∗^; *d* = 1.93	5.24 (1.71) −0.13 (0.85)	4.87 (1.89) −0.32 (0.94)	*U* = 822; *p* = 0.333; *d* = 0.21
Reading with lexical decision	6.17 (1.62) 0.34 (0.81)	2.55 (0.89) −1.48 (0.44)	*U* = 21; *p* < 0.001^∗^; *d* = 2.46	5.79 (2.06) 0.15 (1.03)	5.09 (2.14) −0.21 (1.07)	*U* = 739; *p* = 0.093; *d* = 0.34
Text reading	5.66 (2.06) 0.08 (1.03)	2.30 (1.03) −1.60 (0.52)	*U* = 96; *p* < 0.001^∗^; *d* = 1.81	5.30 (2.54) −0.10 (1.27)	4.65 (2.21) −0.43 (1.10)	*U* = 785; *p* = 0.284; *d* = 0.28
Text writing	4.94 (2.01) −0.28 (1.01)	1.95 (1.36) −1.78 (0.68)	*U* = 155; *p* < 0.001^∗^; *d* = 1.60	4.47 (2.35) −0.52 (1.18)	4.15 (2.22) −0.68 (1.11)	*U* = 881; *p* = 0.642; *d* = 0.14
Word writing	4.48 (1.98) −0.51 (0.99)	2.00 (1.26) −1.75 (0.63)	*U* = 194; *p* < 0.001^∗^; *d* = 1.36	4.18 (2.32) −0.66 (1.16)	3.76 (1.98) −0.87 (0.99)	*U* = 887; *p* = 0.679; *d* = 0.20
Phoneme deletion	5.35 (1.86) −0.08 (0.93)	3.40 (2.11) −1.05 (1.06)	*U* = 346; *p* = 0.001^∗^; *d* = 1.03	5.24 (1.96) −0.13 (0.98)	4.71 (2.14) −0.4 (1.07)	*U* = 777; *p* = 0.173; *d* = 0.26
Battery of phonological tasks	5.19 (2.02) −0.16 (1.01)	3.55 (1.54) −0.98 (0.77)	*U* = 338; *p* < 0.001^∗^; *d* = 0.86	5.15 (1.48) −0.18 (0.74)	4.62 (2.31) −0.44 (1.15)	*U* = 832; *p* = 0.379; *d* = 0.26
Pseudo-word repetition	5.19 (1.67) −0.16 (0.84)	3.45 (1.79) −1.03 (0.90)	*U* = 327; *p* < 0.001^∗^; *d* = 1.04	4.68 (1.59) −0.41 (0.80)	4.87 (1.99) −0.32 (1.00)	*U* = 836; *p* = 0.397; *d* = 0.10

*^∗^Remains significant after Bonferroni correction for nine comparisons. Sten scores are reported (population *M* = 5.5, *SD* = 2.0) followed by sten scores transformed to *z* scores (population *M* = 0.0, *SD* = 1.0).*

#### In-Scanner Performance

Children with dyslexia underperformed typical readers in accuracy in the in-scanner Rhyme task, but only at the first time point (see Table 3). This difference was also significant when the group was restricted to children with a familial history of dyslexia (see Supplementary Table S4). The two groups did not differ either in accuracy in the Voice task, or reaction times in the in-scanner tasks.

**TABLE 3:** fMRI experiment: whole sample (TR, typical readers; DR, children with dyslexia; FHD−, children without a familial history of dyslexia; FHD+, children with a familial history of dyslexia; TP, time point).

	**TR**	**DR**		**FHD+**	**FHD−**	
	***n* = 70**	***n* = 20**		***n* = 55**	***n* = 35**	
Rhyme task: accuracy TP1 (percent of correct responses)	92.25 (12.88)	77.75 (21.73)	*U* = 313; *p* < 0.001^∗^; *d* = 0.96	93.24 (9.45)	86.36 (19.04)	*U* = 733; *p* = 0.077; *d* = 0.43
Voice task: accuracy TP1 (percent of correct responses)	71.74 (20.24)	66.39 (20.28)	*U* = 514; *p* = 0.297; *d* = 0.27	73.22 (18.92)	69.07 (21.01)	*U* = 772; *p* = 0.407; *d* = 0.21
Rhyme task: accuracy TP3 (percent of correct responses)	93.91 (11.14)	90.50 (10.5)	*U* = 513; *p* = 0.066; *d* = 0.31	91.57 (14.94)	94.17 (7.51)	*U* = 899; *p* = 0.681; *d* = 0.24
Voice task: accuracy TP3 (percent of correct responses)	87.75 (13.57)	81.25 (15.72)	*U* = 515; *p* = 0.080; *d* = 0.47	87.29 (12.80)	85.65 (15.20)	*U* = 898; *p* = 0.689; *d* = 0.12
Rhyme task: reaction times TP1 (seconds)	1.71 (0.45)	1.87 (0.39)	*U* = 539; *p* = 0.138; *d* = 0.38	1.61 (0.36)	1.83 (0.47)	*U* = 584; *p* = 0.003; *d* = 0.50
Rhyme task: reaction times TP3 (seconds)	2.04 (0.50)	2.14 (0.51)	*U* = 568; *p* = 0.640; *d* = 0.19	1.97 (0.38)	2.11 (0.56)	*U* = 651; *p* = 0.056; *d* = 0.28
Voice task: reaction times TP1 (seconds)	1.96 (0.60)	1.94 (0.55)	*U* = 654; *p* = 0.723; *d* = 0.04	1.87 (0.61)	2.02 (0.57)	*U* = 802; *p* = 0.228; *d* = 0.25
Voice task: reaction times TP3 (seconds)	2.13 (0.53)	2.19 (0.5)	*U* = 669; *p* = 0.836; *d* = 0.11	2.05 (0.52)	2.2 (0.52)	*U* = 725; *p* = 0.064; *d* = 0.30
Rhyme task: rejected volumes TP1	2.76 (3.94)	3.60 (3.44)	*U* = 559; *p* = 0.148; *d* = 0.22	3.69 (4.44)	2.47 (3.34)	*U* = 841; *p* = 0.288; *d* = 0.32
Rhyme task: rejected volumes TP3	2.64 (3.98)	4.90 (4.60)	*U* = 483; *p* = 0.026; *d* = 0.56	2.46 (3.74)	3.58 (4.45)	*U* = 825; *p* = 0.229; *d* = 0.27
Voice task: rejected volumes TP1	3.29 (5.02)	2.70 (3.87)	*U* = 679; *p* = 0.821; *d* = 0.12	3.57 (5.64)	2.89 (4.43)	*U* = 940; *p* = 0.840; *d* = 0.14
Voice task: rejected volumes TP3	3.00 (4.84)	4.30 (5.37)	*U* = 570; *p* = 0.165; *d* = 0.27	2.63 (4.35)	3.71 (5.32)	*U* = 864; *p* = 0.368; *d* = 0.22

#### fMRI Results

**FIGURE 2:**
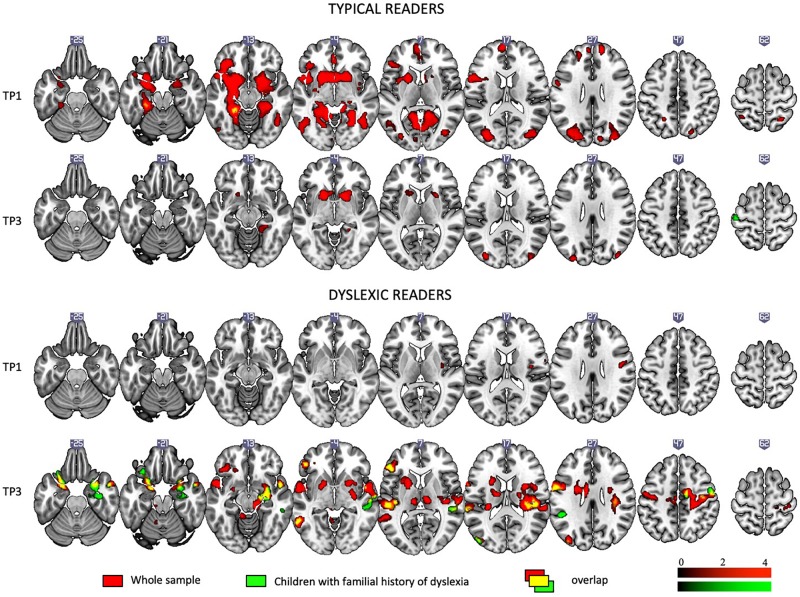
Rhyme – Voice contrast in typical and dyslexic readers at the first and the third time points as revealed by one-sample *t*-tests.

Figure 2 depicts brain activation to Rhyme > Voice (see Experimental Design: fMRI tasks) contrast in typical readers and children with dyslexia at each time point (for details see Supplementary Table S5).

At the first time point, typical readers engaged widespread brain networks including the bilateral inferior frontal areas, the inferior, middle, superior, and anterior temporal areas, the left fusiform gyrus and calcarine sulcus, the cingulate cortex and putamen and caudate (subcortically). At the third time point, activation was restricted to the bilateral putamen, caudate, and occipital areas.

At the first time point, children with dyslexia showed modest activation in the right insula and precentral and postcentral gyri. At the third time point, they showed activation in numerous regions including the bilateral middle, superior temporal, and parietal areas, the bilateral inferior frontal areas, the left insula, the cerebellum (IV, V), and subcortical structures such as the putamen, caudate, amygdala, and hippocampus.

**FIGURE 3:**
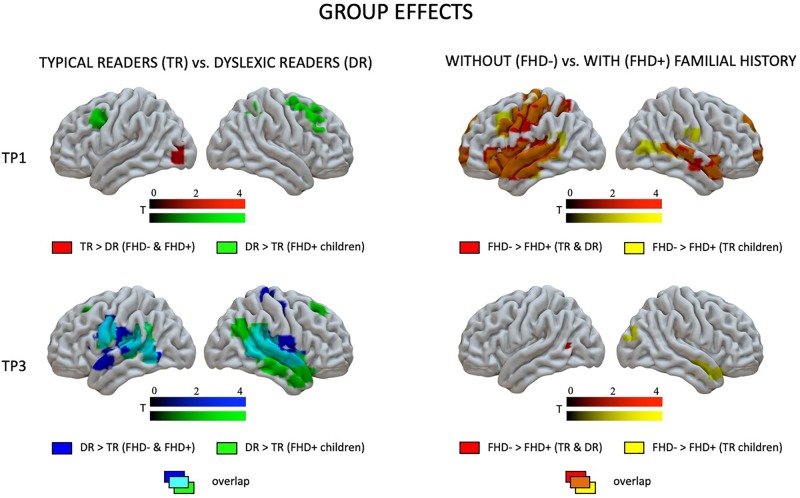
Effects of dyslexia (typical readers vs. dyslexic readers) and familial history of dyslexia (group with familial history of dyslexia vs. children without familial history of dyslexia) at the first and the third time points as revealed by two-sample *t*-tests.

**FIGURE 4:**
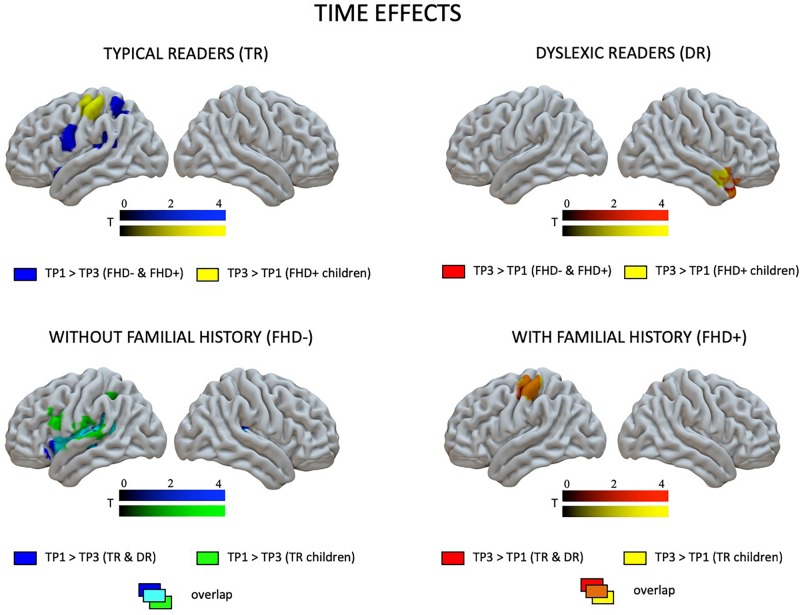
Effects of time (the first vs. the third time point) in children with and without dyslexia and with and without a familial history of dyslexia as revealed by paired *t*-tests.

**FIGURE 5:**
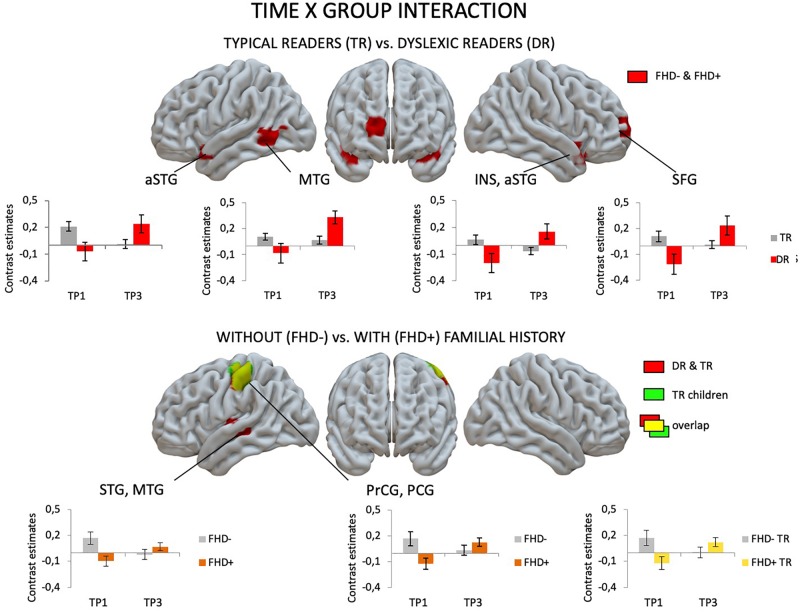
Effects of group (children with dyslexia vs. typical readers; children with a familial history of dyslexia vs. children without a familial history of dyslexia) and time (the first vs. the third time point) interaction as revealed in flexible factorial design.

At the first time point, the only significant difference in brain activation to phonological processing between typical readers and children with dyslexia was in the left middle and inferior occipital gyri, where children with dyslexia had reduced activation compared to typical readers (Figure 3 and Table 4). However, at the third time point, children with dyslexia had higher brain activation than typical readers in several areas. These differences were found mainly in the bilateral superior temporal gyrus (STG), middle temporal gyrus (MTG), Heschl’s gyri (HG), Rolandic operculum and insula, but also in the left supramarginal gyrus (SMG), precentral and postcentral gyri, and subcortically in the right putamen. After 2 years, typically reading children showed a reduction of brain activation in the language regions of the left hemisphere (STG, insula, inferior frontal gyrus: IFG, precentral gyrus: PrCG, superior and inferior parietal lobules: SPL, IPL, hippocampus; Figure 4). In children with dyslexia, brain activation during phonological processing in the right STG and insula increased with time. Diverging developmental trajectories related to literacy acquisition in typical readers and children with dyslexia were confirmed in a time x group interaction present in the bilateral STG, insula, left MTG, and right frontal cortex (see Figure 5 and Table 4).

**TABLE 4:** Significant group and time point effects (first vs. third time point: TP1 vs. TP3) across groups of children with (DR) and without dyslexia (TR).

	**Brain region**	***H***	***x***	***y***	***z***	***t***	***p***	**Voxels**
Group Effects	TP1 TR > DR							
	Middle and Inferior Occipital Gyri	L	−38	−82	−4	3.11	0.001	71
	TP1 DR > TR							
	–							
	TP3 TR > DR							
	–							
	TP3 DR > TR							
	Middle and Superior Temporal Gyri, Rolandic Operculum, Heshl Gyrus, Insula, Postcentral Gyrus	R	32	−32	24	4.96	<0.001	2401
	Cingulate Gyrus	L	−12	−2	30	4.21	<0.001	359
	Superior Temporal Gyrus, Postcentral Gyrus, Heschl Gyrus	L	−60	−30	14	4.10	<0.001	785
	Inferior Parietal Lobule, SupraMarginal Gyrus	L	−46	−42	26	4.05	<0.001	169
	Postcentral and Precentral Gyri, Inferior Frontal Gyrus (oper)	L	−54	0	28	4.05	<0.001	484
	Superior Temporal Gyrus, Insula	R	52	−2	−4	3.57	<0.001	224
	Hippocampus, Putamen	R	30	−12	−12	3.56	<0.001	127
	Middle Temporal Gyrus	L	−52	−48	6	3.46	<0.001	148
	Superior Temporal Gyrus	L	−60	6	−12	3.37	0.001	100
	Precentral Gyrus	R	24	−30	68	3.06	0.001	53
	Insula, Superior Temporal Gyrus	L	−36	−8	−2	3.06	0.001	98
	Precentral Gyrus	R	38	−14	52	2.95	0.002	60
TP Effects	TR TP1 > TP3							
	Precentral Gyrus	L	−54	0	28	3.54	<0.001	102
	Inferior Frontal Gyrus (orb), Insula, Superior Temporal Gyrus	L	−30	6	−18	3.45	<0.001	104
	Superior Parietal Lobule	L	−24	−54	44	3.44	<0.001	59
	Inferior Parietal Lobule	L	−38	−52	62	3.43	0.001	56
	Inferior Frontal Gyrus (oper)	L	−34	12	18	3.33	0.001	60
	Hippocampus	L	−24	−28	0	3.31	0.001	140
	Lingual, Cerebellum (IV, V)	L	−10	−52	0	3.27	0.001	74
	SupraMarginal Gyrus, Superior Temporal Gyrus	L	−44	−42	24	3.21	0.001	64
	DR TP3 > TP1							
	Superior Temporal Gyrus, Insula	R	38	22	−26	4.11	<0.001	114
Interaction	TR TP1 > TP3 and DR TP3 > TP1							
	Superior and Medial Frontal Gyri	R	16	62	8	3.46	<0.001	88
	Middle and Inferior Temporal Gyri	L	−58	−62	0	3.34	0.001	135
	Insula, Superior Temporal Gyrus	R	42	12	−14	3.25	0.001	61
	Superior Temporal Gyrus	L	−30	6	−22	2.91	0.002	61

*Group effects were tested with one-way ANOVA, TP effects were tested with paired *t*-tests, Group and TP interaction was tested with flexible factorial design.*

The pattern of effects seen in typical readers and children with dyslexia was different when the sample was restricted to children with a familial history of dyslexia. This is due to generally reduced brain activation to Rhyme > Voice in typical readers at risk for dyslexia that was restricted to the left fusiform (TP1) and postcentral gyrus (TP3) (for details see Supplementary Table S5). This is why, at the first time point, children with a familial history of dyslexia who later developed reading impairment had higher brain activation in the bilateral middle frontal gyri as well as in the right inferior parietal lobule than children who became typical readers. Again, at the third time point, children with a familial history of dyslexia who developed dyslexia had higher brain activation than children who did not develop any reading impairment. This activation was more extensive than at the first time point and included the bilateral STG, MTG, SMG, Heschl’s gyri, Rolandic operculum, left precentral and postcentral gyri, bilateral putamen (subcortically), left thalamus, right amygdala, and hippocampus (see Figure 3 and Supplementary Table S7). Time effects in typical readers with a familial history of dyslexia were restricted to higher activation in the left precentral and postcentral gyri at the third compared to the first time point. Increased activation in the right STG was observed (Figure 5) in children with dyslexia from the familial risk group just as was observed for the whole sample of children with dyslexia. However, in the case of children with a familial history of dyslexia, the interaction between time and group (typical readers vs. dyslexic readers) was not significant.

### Effects of Familial History of Dyslexia

#### Behavioral Results

**FIGURE 6:**
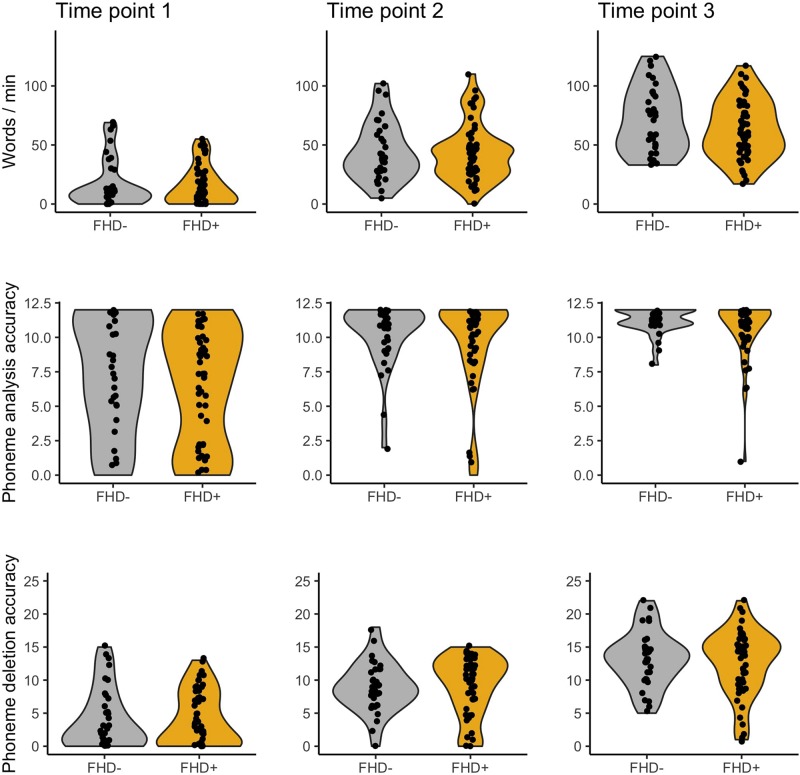
Word reading, phoneme analysis, and phoneme deletion of children without (FHD–) and with a familial history of dyslexia (FHD+) across three time points.

No significant differences in early reading and reading related tests were observed between children with and without a familial history of dyslexia (see Table 1 and Figure 6), although the groups differed in parental scores in the Adult Reading History Questionnaire and in home literacy environment. These two groups also had similar performance in all tests belonging to the dyslexia diagnosis battery (see Table 2) run after 2 years of school education. This pattern of lack of between group results remained unchanged when the sample was restricted to typical readers (see Supplementary Tables S2, S3).

#### In-Scanner Performance

No significant differences in accuracy in in-scanner tasks were observed between children with and without a familial history of dyslexia (see Table 3). However, children with a familial history of dyslexia were slower in the Rhyme task at the first time point. On the other hand, this difference appeared only at the trend level in typical readers (see Supplementary Table S4).

#### fMRI Results

**FIGURE 7:**
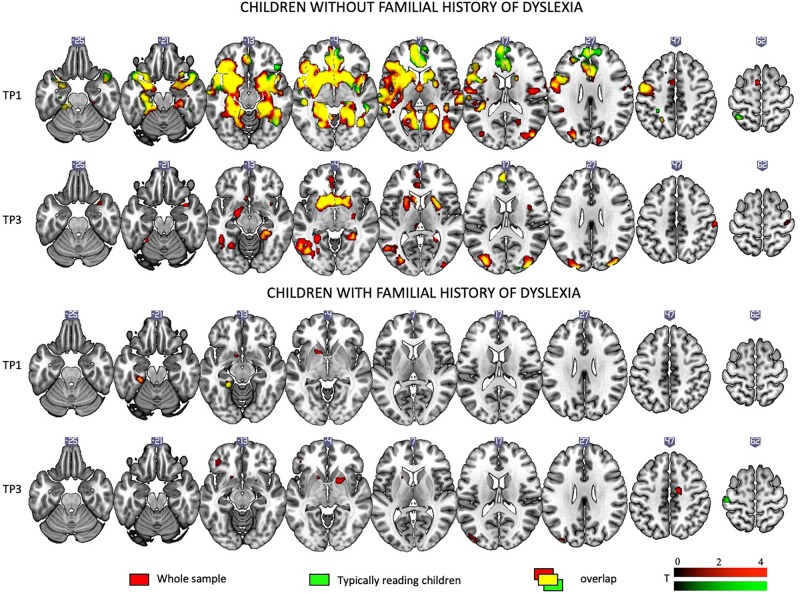
Rhyme – Voice contrast in children with and without a familial history of dyslexia at the first and the third time points as revealed by one-sample *t*-tests.

Figure 7 depicts brain activation to Rhyme > Voice (see Experimental Design: fMRI tasks) for children with and without a familial history of dyslexia (for details see Supplementary Table S6).

When the total sample was split into children with and without a familial history of dyslexia, irrespective of a later dyslexia diagnosis, at the first time point, children with a familial history of dyslexia showed activations only in left subcortical areas. Reduced activation persisted at the third time point and included the bilateral occipital areas and putamen.

Children without a familial history of dyslexia at the first time point engaged an extensive network including the bilateral middle, superior temporal, and parietal areas, the bilateral inferior frontal areas, insula, occipital areas, and the fusiform gyrus as well as subcortical structures such as the *inter alia* putamen, caudate, amygdala, hippocampus, and thalamus. At TP3, activation was similar to TP1 and present mainly in the middle and superior occipital areas, the inferior frontal areas, and the left fusiform and subcortical structures (for details see Supplementary Table S6).

Comparing children with a familial history of dyslexia to those without showed reduced brain activation in widespread brain areas. These areas included the bilateral STG, MTG, Insula, left IFG and superior frontal gyrus (SFG), SPL, IPL, right lingual gyrus (LG) and cerebellum (III, IV, V), left postcentral and precentral gyri, and left thalamus (subcortically). After 2 years, children with a familial history of dyslexia still presented reduced brain activation but this was restricted to the left MTG, caudate and bilateral anterior cingulate (see Figure 3 and Table 5). In children without a familial history of dyslexia, a reduction of brain activation was seen with time, especially in left hemispheric areas. These included the STG, IFG, SMG, insula and bilaterally in the hippocampi. Children with a familial history of dyslexia showed an increase in activity over time in the left postcentral and precentral gyri (see Figure 4). An interaction between group and time was present in left precentral and postcentral gyri as well as in the left STG and MTG (see Figure 5).

**TABLE 5:** Significant group and time point effects across groups of children with (FHD+) and without a familial history of dyslexia (FHD−).

	**Brain region**	***H***	***x***	***y***	***z***	***t***	***p***	**Voxels**
Group Effects	TP1 FHD- > FHD +							
	Middle and Superior Temporal Gyri, Inferior Frontal Gyrus (orb, tri), Rolandic Operculum	L	−46	16	−14	4.88	<0.001	4108
	Insula, Middle and Superior Temporal Gyri	R	42	8	−12	4.21	<0.001	434
	Superior and Medial Frontal Gyri	L	−14	64	2	3.88	<0.001	234
	Thalamus	L	−2	−4	2	3.56	<0.001	129
	Superior and Inferior Parietal, Postcentral Gyrus	L	−26	−50	56	3.46	<0.001	148
	Postcentral and Precentral Gyri	L	−56	0	38	3.46	<0.001	997
	Superior Frontal Gyrus	L	−2	50	40	3.28	0.001	156
	Precentral Gyrus	L	−18	−18	70	3.28	0.001	63
	Middle Temporal Gyrus, Hippocampus	R	30	−38	6	3.24	0.001	152
	Middle and Superior Temporal Gyri	R	54	−14	−12	3.23	0.001	182
	Cerebellum (III, IV, V), Lingual	R	12	−32	−12	3.16	0.001	109
	Medial frontal (sup)	R	16	64	0	3.15	0.001	85
	Anterior Cingulate (L, R)	L, R	−36	−26	16	3.00	0.002	53
	Rolandic Operculum, Insula	L	4	36	0	2.86	0.003	59
	TP1 FHD + > FHD-							
	–							
	TP3 FHD− > FHD +							
	Caudate (L), Anterior Cingulate (L, R)	L, R	−6	20	−6	3.72	<0.001	178
	Anterior Cingulate	L	−4	38	8	3.15	0.001	67
	Middle Temporal Gyrus	L	−40	−54	0	3.08	0.001	64
	TP3 FHD + > FHD−							
	–							
TP Effects	FHD− TP1 > TP3							
	Insula, Superior Temporal Gyrus, Rolandic Operculum, Inferior Frontal Gyrus (oper), Amygdala	L	−40	−12	8	3.97	<0.001	505
	Hippocampus	R	36	−34	4	3.72	<0.001	147
	Superior Temporal Gyrus, SupraMarginal Gyrus	L	−44	−42	24	3.63	<0.001	200
	Hippocampus	L	−26	−30	−2	3.50	0.001	71
	Insula, Inferior Frontal (orb and tri)	L	−38	18	−10	3.20	0.001	51
	Insula, Superior Temporal Gyrus, Rolandic Operculum, Inferior Frontal Gyrus (oper), Amygdala							
	FHD + TP3 > TP1							
	Postcentral and Precentral Gyri	L	−42	−26	66	3.25	0.001	165
Interaction	FHD− TP1 > TP3 and FHD + TP3 > TP1							
	Postcentral and Precentral Gyri	L	−44	−22	58	3.28	0.001	178
	Superior and Middle Temporal Gyri	L	−46	−26	0	3.06	0.001	52

*Group effects were tested with one-way ANOVA, TP effects were tested with paired *t*-tests, Group and TP interaction was tested with flexible factorial design.*

When the sample of children was restricted to typical readers, significant differences were still observed between children with and without a familial history of dyslexia. Again, at both time points, children with a familial history of dyslexia had reduced brain activation compared to children without dyslexia in families (see Supplementary Table S6). At the first time point, group differences were found in the bilateral STG, MTG, insula, left precentral and postcentral gyri, IFG, MFG and SFG, right LG and cerebellum (IV, V), and left thalamus (subcortically). At the third time point, the differences were reduced to the right STG, MTG, left caudate and bilateral putamen (see Supplementary Table S8 and Figure 3). While children without a familial history of dyslexia showed a reduction of brain activation with time in the left hemispheric language areas (STG, IFG, SMG, HG, Insula, Rolandic Operculum) and bilaterally in the hippocampi, children with a familial history of dyslexia increased activation in the left precentral and postcentral gyri (see Figure 4). The interaction between group and time was significant in the left precentral and postcentral gyri (see Figure 5).

## Discussion

### Effects of Dyslexia

In the present study, we investigated longitudinally how neural correlates of phonological processing change during the two first years of reading instruction in typical readers and in children with dyslexia. We also examined these effects in children with and without familial history of dyslexia, irrespectively of dyslexia itself, which will be discussed in the following section.

At the behavioral level, children with dyslexia performed lower than typical readers in reading, phonological awareness, and rapid automatized naming tests at each time point, even at the very beginning of education. The behavioral differences were stable when the sample was restricted to children with a familial history of dyslexia. Similar differences between typical and poor readers have also been reported at a very early stage of literacy development (kindergarden) in other orthographies (Czech and Slovak: Moll et al., 2016, Dutch: Dandache et al., 2014, Finnish: Torppa et al., 2010, German: Schneider et al., 2000, English: Gallagher et al., 2000, for meta-analysis see: Snowling and Melby-Lervåg, 2016). This means that in many orthographies, including Polish, behavioral differences between future poor and fluent readers may be observed much earlier than after several years of reading instruction.

In terms of BOLD activations as registered by fMRI, the pattern of differences observed between typical and dyslexic readers at the beginning of education was different depending on whether the analyses included the whole sample or only children with a familial risk for dyslexia. Reduced activation of the left visual cortex in dyslexics was present only when all children were included in the analysis (consistent with previous fMRI results in visual and orthographic processing tasks, e.g., Dehaene et al., 2010; Boros et al., 2016; Cao et al., 2018). It was no longer significant when the group was restricted to children with a familial history of dyslexia. When the group was restricted to children with a familial history of dyslexia, hyperactivation in the dyslexic group was observed, which included mainly the right fronto-parietal regions. The differences between dyslexia effects in the total sample and in the analyses restricted to children with a familial risk seem to be primarily related to a large reduction in activation of the phonological network in typical readers with a familial risk for dyslexia.

At the third time point, 2 years after the first time point, children with dyslexia showed increased activation compared to typical readers in the bilateral temporal cortices including the auditory cortex, as well as in the left supramarginal and precentral and postcentral gyri, and in the putamen (subcortically). These areas are typically associated with the neural phonological network (for e.g., Brennan et al., 2013) and were also employed by typical readers at the first time point. The observed overactivation might support the notion that children with dyslexia present a delay in the development of their phonological network (Raschle et al., 2011; Morken et al., 2017), as after 2 years of education they activate the regions that typical readers activated at an earlier stage of development. There is, however, a debate on the issue as to whether dyslexia is a developmental delay or a deficit with an altered developmental pathway. With respect to phonological skills, a cross-sectional study in dyslexic children using a developmental trajectory method (Thomas et al., 2009) revealed a delayed trajectory for phonological short term memory and RAN, but showed an atypical trajectory for phonological awareness (Kuppen and Goswami, 2016). An atypical rather than delayed phonological brain network in dyslexia was also found in a cross-sectional fMRI study where dyslexic children exhibited reduced activation during a rhyme judgment task in the bilateral temporo-parietal and frontal cortex relative to both age-matched and reading-matched children (Hoeft et al., 2006). However, cross-sectional studies cannot definitively distinguish between atypical and delayed development of the phonological brain network. More longitudinal studies are needed to resolve this debate. As our data covers only the first 2 years of education, the current set of data does not allow us to predict what happens with the phonological network after this period. Nor can we say whether or not the activations observed in the dyslexic group would begin to resemble those of typical readers or whether their behavior over time will be the same.

At the very first stage of literacy acquisition, typical readers engaged not only structures typically involved in phonological processing (bilateral superior and middle temporal gyri, left IFG) but also those involved in semantic analysis of words (anterior temporal areas) and in movement planning (premotor and motor areas, caudate, putamen). The reduced activation at the third time point in the phonological network, especially in the left perisylvian areas, suggests that with growing reading experience or time, typical readers automatize phonological processing and therefore the neural circuitry becomes more specialized (Pugh et al., 2013; Dębska et al., 2016; Yu et al., 2018). This finding contrasts with the age-related increases in activation found in cross-sectional studies (Cone et al., 2008; Brennan et al., 2013). These reductions are more pronounced (not restricted to left IPL) than what was observed in a previous longitudinal study of typical readers (Yu et al., 2018), probably because of a wider time period between the two time points and a larger sample size in the current study.

After 2 years, children with dyslexia (irrespective of familial history status) showed increased brain activation, and they engaged the right hemisphere superior temporal cortex. The right hemisphere is commonly employed during reading by typical beginning readers (Waldie and Mosley, 2000) and its activity declines as literacy develops (Shaywitz et al., 2007). Previous studies reported compensatory shifts to right hemisphere in dyslexia in terms of activation increases (Shaywitz et al., 2007; Simos et al., 2007).

The interaction of group and time observed in the bilateral STG, insula, left MTG, and right frontal cortex supports the hypothesis of a delay in the development of phonological structures in dyslexic readers (Raschle et al., 2011; Morken et al., 2017). These brain areas seem to be involved in early phonological processing related to low reading skills, as they were employed by typical readers only at the beginning of literacy, and by dyslexic children that are 2 years older. Lack of interaction when the group is restricted to children with a familial history of dyslexia suggests some additive influence of both factors. As the interaction appears only if both children with and without a familial history of dyslexia are included, perhaps it is driven by activations of children without a familial risk who reshape the phonological network more clearly.

### Effects of Familial History of Dyslexia

In contrast to previous studies, no behavioral differences were found between children with and without a familial history of dyslexia at any time point, even though the groups differed in home literacy environment and parental reading history. Previously, in the relatively transparent Norwegian language, behavioral performance of children with and without a familial history of reading impairment started to differ in the second grade (Specht et al., 2009), though there were no differences at earlier stages. In the more opaque English language, the gap was visible even at earlier stages of education (Raschle et al., 2012). The effect of a familial history of dyslexia may depend on the depth of the orthography. As shallow orthographies are more easily acquired, differences between readers with and without a familial history of dyslexia are less visible, whereas more demanding opaque orthographies lead to earlier observable differences. However, this hypothesis was not supported by a recent meta-analysis (Snowling and Melby-Lervåg, 2016), where no effect of language transparency was revealed. In a meta-analysis based on two to nine studies, depending on the analyzed cognitive skill, the authors found that typical readers with a familial history of dyslexia overcome early delays in vocabulary, grammar, and phonological skills by the time of schooling. However, they still present poorer performance in word and non-word decoding and spelling. This was not the case in our study.

It is plausible that we have overestimated the number of children with a familial history of dyslexia as the group selection was based on quite liberal criteria. Likewise, perhaps fewer children were at severe risk of dyslexia. In the analyzed sample, 27.27% of children with familial history of dyslexia received dyslexia diagnosis, in contrast to the 45% reported in a recent meta-analysis (Snowling and Melby-Lervåg, 2016). On the other hand, the relatively high reading level of children with familial risk of dyslexia may have resulted from the presence of several protective factors (Eklund et al., 2013). These children not only scored moderately to highly in cognitive tests, but also came from well-educated families of relatively high socioeconomic status. What is more, their parents were probably concerned about reading development of their children and perhaps provided some additional training, which we did not control for. Also, we have interpreted the risk of dyslexia as a dichotomous variable, although it can also be understood as a continuum (Snowling et al., 2003). Perhaps a stricter split point would result in between-group differences. However, the criterion we used in the current study have previously been applied in research on other languages (Lefly and Pennington, 2000) and have been approved for use in Polish (Bogdanowicz et al., 2015).

Consistent hypoactivation was present for children with a familial history of dyslexia compared to children without such a history, and included the bilateral superior and middle temporal cortex, left precentral and postcentral gyri, left inferior frontal areas, right cerebellum and visual cortex, as well as left thalamus. This pattern is in line with previous studies comparing pre-readers with and without a familial history of dyslexia (Raschle et al., 2012) and beginning readers (Dębska et al., 2016). We show for the first time that a familial history of dyslexia modifies brain activation during phonological processing even in typical readers, not only children diagnosed later with dyslexia.

The hypoactivation in the group at risk of dyslexia was visible at the third time point. After 2 years of education, children with a familial history of dyslexia compared to children without such history (even when restricted only to typical readers) still show hypoactivations in the temporal cortex and subcortically in the caudate and putamen, areas which have been implicated in phonological processing (Georgiewa et al., 1999; Cone et al., 2008). Even though cognitive and reading-related skills are still largely developing at school age, having a familial risk for dyslexia can be considered a more stable risk factor also at the neural level. It may also be interpreted as an indirect proxy for genotype-related properties, in this case resulting in hypoactivation of language structures during phonological processing. Having a familial risk rather than dyslexia itself is also related to atypical planum temporale asymmetry important in speech processing (Vanderauwera et al., 2017) as well as basic auditory processing deficits at the brain level (Hakvoort et al., 2015). Though we are still far from seeing the fuller picture, we provide additional evidence that some cerebral anomalies might be present early in development and might be related to familial risk, irrespective of the later reading outcome.

Typical readers with a familial history of dyslexia show a different neurodevelopmental trajectory, as revealed by group and time interaction in typical readers. With literacy development, typical readers with a familial history of dyslexia show increased engagement of the left precentral and postcentral gyri, activated in covert articulation (Price, 2010), whereas children without a familial history of dyslexia present a decrease in activation of these areas. Perhaps these brain regions support phonological processing in children with a familial history of dyslexia who happen not to develop reading impairment by relying more on covert articulation than on orthographic processing.

### Limitations

The main limitation of the project is that, despite a relatively high number of recruited participants, we did not find enough children without familial risk of reading impairment who developed dyslexia to run analyses in a full 2 × 2 model. There were only five children without familial risk of dyslexia identified as having a reading impairment. This number was too small to include this group in analyses. Therefore, instead of including effects of dyslexia and familial history of dyslexia in one model, we decided to run the analyses in a model previously applied in a study of white matter dynamics (Vanderauwera et al., 2017) having a similar group of participants.

Moreover, the age range of our sample was quite wide at each time point. As described in the Section “Materials and Method,” this was a result of ongoing educational reform in Poland. The sample reflected the group of children starting formal education in Poland in the years of our study. However, due to the large variability in the ages of children starting schooling, the oldest participants of the first time point (kindergartens or first graders) were almost as old as the youngest participants of the third time point (second or third graders). Thus, we were unable to include age as a factor in the analyses, and our focus was on educational experience.

Additionally, due to the short history of diagnosing dyslexia in Poland, it was impossible to select children with a familial history of dyslexia only on the basis of formal diagnosis of parents and/or siblings. Although some of the parents presented a full spectrum of dyslexia symptoms, they had not been diagnosed with reading impairment due to the fact that when they attended school, dyslexia was not formally diagnosed in Poland. Instead of relying solely on a formal diagnosis, we were forced to apply a questionnaire measure of familial risk of dyslexia, which was perhaps biased by the memories of the parents. Relying on this questionnaire may have had an impact on the lack of behavioral differences observed between children selected as having a familial risk of dyslexia and children without such risk.

Although we tried to include as many participants of various socio-economic status as possible, the variance in socio-economic status was relatively small and children diagnosed with dyslexia had slightly (although statistically insignificantly) lower socio-economic status than typical readers. The majority of the participants of the current study lived in Warsaw and had parents of high educational and socio-economic status. As the study was longitudinal and quite demanding for participants, it was impossible to include participants from rural areas of Poland.

Finally, for the results reported in the current paper we used *p* < 0.005 uncorrected and a cluster size of 50 voxels. Though the results were not corrected for multiple comparisons, in pediatric neuroimaging more liberal thresholds are usually used (usually not corrected), especially in children this young. This is because children show reduced signal-to noise ratios (see e.g., Thomason et al., 2005), time shifted hemodynamic response functions, increased movement throughout the scan, and reduced overall compliance. Additionally, less data is usually acquired to keep scanning time as low as possible. Indeed an uncorrected thresholds of *p* < 0.005 are commonly reported in fMRI studies on children (Raschle et al., 2012, 2014; Langer et al., 2015; Saygin et al., 2016). We believe that applying standards common for adult participants’ studies may be possible upon solving some technical issues related to scanning (e.g., child appropriate head coils, age appropriate hemodynamic response functions in standard analysis packages etc.).

## Conclusion

We conclude that the phonological brain network undergoes reorganization during the first 2 years of reading acquisition and that this process proceeds differently depending on the presence of a familial history of dyslexia and reading impairment. Typical readers without risk for dyslexia activate structures responsible for phonological processing already at the beginning of literacy. In this group, reduced brain activation over time during phonological processing is plausibly due to automatization of phonological skills. Children who develop reading impairment present a kind of delay in the development of language and, in particular, phonological structures such as the bilateral STG, left MTG, right insula, and right frontal cortex, where we observed time and group interaction. Finally, typical readers with familial risk of dyslexia also present an atypical development of the neural phonological structures, visible both at the beginning of reading instruction and 2 years later. These children used a presumably efficient neural mechanism of phonological processing, based on the activation of the precentral and postcentral gyri, and achieved a typical level of phonological awareness possibly through the use of silent articulation.

## Data Availability Statement

The datasets generated for this study are available on request to the corresponding author.

## Ethics Statement

The studies involving human participants were reviewed and approved by the Warsaw University Ethical Committee. Written informed consent to participate in this study was provided by the participants’ legal guardian/next of kin.

## Author Contributions

MŁ and KJ wrote the manuscript. AG, AD, and KC contributed to the writing process. MŁ, KJ, KC, and AD analyzed and interpreted the data and prepared the tables and figures. MŁ, AB, KC, AD, and AŻ acquired the data. AM and AG additionally contributed to conception and design of the study. All authors read and revised the article.

## Conflict of Interest

The authors declare that the research was conducted in the absence of any commercial or financial relationships that could be construed as a potential conflict of interest.
