# Supplementary material for: Children With Dyslexia and Familial Risk for Dyslexia Present Atypical Development of the Neuronal Phonological Network
Source: Front Neurosci. 2019 Nov 29;13:1287. doi: 10.3389/fnins.2019.01287 (PMC6895138; doi:10.3389/fnins.2019.01287)
Supplement: Supplementary file 1 [file Data_Sheet_1.docx]

# Supplementary material

Table S1. The stimuli material used in the fMRI tasks.

| Task | Item | Polish words | | English translation | | Correct response |
| --- | --- | --- | --- | --- | --- | --- |
| Rhyme | 1 | kot | płot | cat | fence | 1 |
| Rhyme | 2 | trawa | lawa | grass | lava | 1 |
| Rhyme | 3 | czapka | lody | cap | ice cream | 0 |
| Rhyme | 4 | róża | burza | rose | storm | 1 |
| Rhyme | 5 | stos | łapka | pile | paw | 0 |
| Rhyme | 6 | schody | boisko | stairs | playground | 0 |
| Rhyme | 7 | taczka | paczka | barrow | package | 1 |
| Rhyme | 8 | słoń | dłoń | elephant | hand | 1 |
| Rhyme | 9 | broda | gruszka | beard | pear | 0 |
| Rhyme | 10 | sowa | krowa | owl | cow | 1 |
| Rhyme | 11 | ptaki | woda | birds | water | 0 |
| Rhyme | 12 | czoło | koło | forehead | circle | 1 |
| Rhyme | 13 | dach | maki | roof | poppies | 0 |
| Rhyme | 14 | las | pas | forest | belt | 1 |
| Rhyme | 15 | osa | pietruszka | wasp | parsley | 0 |
| Rhyme | 16 | piach | kosa | sand | scythe | 0 |
| Rhyme | 17 | rama | brama | frame | gate | 1 |
| Rhyme | 18 | nosze | ognisko | litter | campfire | 0 |
| Rhyme | 19 | nos | kalosze | nose | rain boots | 0 |
| Rhyme | 20 | półka | bułka | shelf | bread roll | 1 |
| Voice | 1 | łapka | paczka | paw | package | 1 |
| Voice | 2 | czapka | róża | cap | rose | 0 |
| Voice | 3 | piach | stos | sand | pile | 1 |
| Voice | 4 | lawa | kot | lava | cat | 1 |
| Voice | 5 | burza | płot | storm | fence | 0 |
| Voice | 6 | nos | dach | nose | roof | 0 |
| Voice | 7 | pietruszka | las | parsley | forest | 1 |
| Voice | 8 | maki | krowa | poppies | cow | 1 |
| Voice | 9 | bułka | schody | bread roll | stairs | 0 |
| Voice | 10 | woda | sowa | water | owl | 1 |
| Voice | 11 | pas | gruszka | belt | pear | 1 |
| Voice | 12 | nosze | brama | litter | gate | 0 |
| Voice | 13 | słoń | trawa | elephant | grass | 1 |
| Voice | 14 | krowa | osa | cow | wasp | 0 |
| Voice | 15 | rama | kosa | frame | scythe | 1 |
| Voice | 16 | dłoń | ognisko | hand | campfire | 0 |
| Voice | 17 | kalosze | lody | rain boots | ice cream | 0 |
| Voice | 18 | koło | boisko | circle | playground | 1 |
| Voice | 19 | broda | taczka | beard | barrow | 0 |
| Voice | 20 | ptaki | czoło | birds | forehead | 0 |

The dyslexic group included 15 children with familial history of dyslexia and 5 children without familial history of dyslexia, whereas the group of typical readers (n = 70) included 40 children with familial history of dyslexia and 30 children without such risk factor

Table S2. Participants’ characteristics: children with familial history of dyslexia and typical readers (TR: typical readers, DR: children with dyslexia, FHD-: children without familial history of dyslexia, FHD+: children with familial history of dyslexia; TP: time point; ARHQ: Adult Reading History Questionnaire)

|  | **FHD+** | |  | **TR** | |  |
| --- | --- | --- | --- | --- | --- | --- |
|  | **TR** | **DR** |  | **FHD-** | **FHD+** |  |
|  | n = 40 | n = 15 |  | n = 30 | n = 40 |  |
| **Age at TP1 (years)** | 6.95 (0.52) | 6.89 (0.41) | U = 282; p = .727;  95% CI DV = [-0.24 ; 0.36]; Cohen's d = 0.13 | 6.92 (0.47) | 6.95 (0.52) | t(68) = -0.288; p = .774;  95% CI DV = [-0.27 ; 0.21]; Cohen's d = 0.07 |
| **Age at TP3 (years)** | 8.98 (0.51) | 8.93 (0.40) | U = 283; p = .741;  95% CI DV = [-0.24 ; 0.34]; Cohen's d = 0.11 | 8.97 (0.51) | 8.98 (0.51) | t(68) = -0.051; p = .960;  95% CI DV = [-0.25 ; 0.24]; Cohen's d = 0.01 |
| **Socioeconomic status** | 49.26 (11.08) | 39.05 (14.03) | U = 170; p = .014;  95% CI DV = [2.97 ; 17.46]; Cohen's d = 0.87 | 50.99 (8.35) | 49.26 (11.08) | t(68) = 0.716; p = .477;  95% CI DV = [-3.09 ; 6.55]; Cohen's d = 0.18 |
| **ARHQ mother** | 37.6 (13.46) | 39.27 (16.04) | U = 298; p = .970;  95% CI DV = [-10.28 ; 6.95]; Cohen's d = 0.12 | 22.43 (7.89) | 37.60 (13.46) | t(68) = -5.497; p < .001*;  95% CI DV = [-20.67 ; -9.66]; Cohen's d = 1.35 |
| **ARHQ father** | 40.32 (15.27) | 47.83 (15.39) | U = 153; p = .088;  95% CI DV = [-17.7 ; 2.67]; Cohen's d = 0.50 | 24.70 (7.74) | 40.32 (15.27) | t(63) = -4.878; p < .001*;  95% CI DV = [-22.01 ; -9.22]; Cohen's d = 1.25 |
| **Home literacy** | 35.44 (5.96) | 31.14 (4.75) | U = 147; p = .011;  95% CI DV = [0.74 ; 7.85]; Cohen's d = 0.77 | 37.55 (5.90) | 35.44 (5.96) | t(66) = 1.454; p = .151;  95% CI DV = [-0.79 ; 5.02]; Cohen's d = 0.36 |
| **Number of letters known at TP1** | 52.40 (15.29) | 31.00 (18.78) | U = 100; p < .001*  95% CI DV = [11.51 ; 31.29]; Cohen's d = 1.34 | 49.97 (14.92) | 52.4 (15.29) | t(68) = -0.666; p = .508;  95% CI DV = [-9.73 ; 4.86]; Cohen's d = 0.16 |
| **Number of letters known at TP2** | 63.00 (2.98) | 60.13 (7.43) | U = 140; p < .001*;  95% CI DV = [0.08 ; 5.66]; Cohen's d = 0.64 | 63.10 (1.95) | 63.00 (2.98) | t(68) = 0.16; p = .874; 9  5% CI DV = [-1.15 ; 1.35]; Cohen's d = 0.04 |
| **Raven Matrices IQ (sten)** | 7.73 (1.26) | 6.6 0(1.55) | U = 176; p = .015;  95% CI DV = [0.31 ; 1.94]; Cohen's d = 0.86 | 7.67 (1.03) | 7.73 (1.26) | t(68) = -0.207; p = .837;  95% CI DV = [-0.62 ; 0.5]; Cohen's d = 0.05 |
| **WISC-R IQ** | 123.6 (11.77) | 113.53 (13.07) | U = 166; p = .011;  95% CI DV = [2.7 ; 17.43]; Cohen's d = 0.85 | 123.41 (13.67) | 123.6 (11.77) | t(67) = -0.061; p = .952;  95% CI DV = [-6.32 ; 5.95]; Cohen's d = 0.02 |
| **WISC-R IQ: verbal scale** | 122.82 (13.89) | 111.6 (17.44) | U = 182; p = .025;  95% CI DV = [2.17 ; 20.28]; Cohen's d = 0.77 | 124.76 (15.12) | 122.82 (13.89) | t(67) = 0.55; p = .584;  95% CI DV = [-5.08 ; 8.95]; Cohen's d = 0.14 |
| **WISC-R IQ: non-verbal scale** | 120.05 (10.81) | 113.2 (11.91) | U = 207; p = .077;  95% CI DV = [0.1 ; 13.6]; Cohen's d = 0.63 | 117.86 (12.44) | 120.05 (10.81) | t(67) = -0.779; p = .439;  95% CI DV = [-7.8 ; 3.42]; Cohen's d = 0.19 |
| **Digit span**  **(number of repeated strings)** | 6.55 (1.91) | 5.60 (1.24) | U = 186; p = .023;  95% CI DV = [-0.12 ; 2.02]; Cohen's d = 0.55 | 6.63 (1.61) | 6.55 (1.91) | t(68) = 0.193; p = .847;  95% CI DV = [-0.78 ; 0.94]; Cohen's d = 0.05 |
| **Syllable span**  **(number of repeated strings)** | 8.55 (2.83) | 7.60 (2.32) | U = 230; p = .179;  95% CI DV = [-0.69 ; 2.59]; Cohen's d = 0.36 | 8.97 (2.25) | 8.55 (2.83) | t(68) = 0.664; p = .509;  95% CI DV = [-0.84 ; 1.67]; Cohen's d = 0.16 |
| **Vocabulary at TP1**  **(percentile)** | 77.46 (21.84) | 54.82 (25.63) | U = 59; p = .009;  95% CI DV = [5.56 ; 39.72]; Cohen's d = 1.01 | 74.50 (22.6) | 77.46 (21.84) | t(42) = -0.44; p = .662;  95% CI DV = [-16.52 ; 10.6]; Cohen's d = 0.14 |
| **Word reading TP1**  **(items read / minute)** | 19.68 (16.92) | 3.93 (5.64) | U = 113; p < .001*;  95% CI DV = [6.75 ; 24.73]; Cohen's d = 1.08 | 19.50 (21.13) | 19.68 (16.92) | t(68) = -0.038; p = .969;  95% CI DV = [-9.25 ; 8.9]; Cohen's d = 0.01 |
| **Word reading TP2**  **(items read / minute)** | 51.43 (22.79) | 22.07 (9.97) | U = 55; p < .001*;  95% CI DV = [17.08 ; 41.63]; Cohen's d = 1.48 | 49.00 (22.95) | 51.43 (22.79) | t(68) = -0.439; p = .662;  95% CI DV = [-13.44 ; 8.59]; Cohen's d = 0.11 |
| **Word reading TP3**  **(items read / minute)** | 73.73 (19.78) | 34.93 (9.82) | U = 9; p < .001*;  95% CI DV = [28.04 ; 49.54]; Cohen's d = 2.23 | 74.79 (28.58) | 73.73 (19.78) | t(67) = 0.184; p = .855;  95% CI DV = [-10.55 ; 12.68]; Cohen's d = 0.05 |
| **Pseudo-word reading TP1**  **(items read / minute)** | 16.2 (13.55) | 3.33 (5.15) | U = 109; p < .001*;  95% CI DV = [5.63 ; 20.11]; Cohen's d = 1.10 | 15.24 (13.42) | 16.20 (13.55) | t(67) = -0.291; p = .772;  95% CI DV = [-7.53 ; 5.61]; Cohen's d = 0.07 |
| **Pseudo-word reading TP2**  **(items read / minute)** | 33.3 (10.19) | 18.67 (7.94) | U = 73; p < .001*;  95% CI DV = [8.78 ; 20.49]; Cohen's d = 1.55 | 33.40 (10.4) | 33.30 (10.19) | t(68) = 0.04; p = .968;  95% CI DV = [-4.85 ; 5.05]; Cohen's d = 0.01 |
| **Pseudo-word reading TP3**  **(items read / minute)** | 41.95 (9.7) | 26.67 (6.8) | U = 52; p < .001*;  95% CI DV = [9.8 ; 20.76]; Cohen's d = 1.72 | 42.07 (13.2) | 41.95 (9.7) | t(67) = 0.043; p = .966;  95% CI DV = [-5.38 ; 5.62]; Cohen's d = 0.01 |
| **Phoneme analysis TP1**  **(items solved)** | 7.7 (3.96) | 2.87 (3.07) | U = 108; p < .001*;  95% CI DV = [2.56 ; 7.11]; Cohen's d = 1.31 | 7.90 (4.11) | 7.70 (3.96) | t(68) = 0.206; p = .838;  95% CI DV = [-1.74 ; 2.14]; Cohen's d = 0.05 |
| **Phoneme analysis TP2**  **(items solved)** | 9.93 (3.03) | 8.93 (3.96) | U = 265; p = .492;  95% CI DV = [-1.01 ; 3]; Cohen's d = 0.31 | 10.70 (1.77) | 9.93 (3.03) | t(68) = 1.249; p = .216;  95% CI DV = [-0.46 ; 2.01]; Cohen's d = 0.30 |
| **Phoneme analysis TP3**  **(items solved)** | 10.35 (2.89) | 10.2 (2.76) | U = 253; p = .349;  95% CI DV = [-1.58 ; 1.88]; Cohen's d = 0.05 | 10.97 (2.23) | 10.35 (2.89) | t(67) = 0.959; p = .341;  95% CI DV = [-0.67 ; 1.9]; Cohen's d = 0.24 |
| **Phoneme deletion TP1**  **(items solved)** | 5.05 (4.13) | 1.40 (2.64) | U = 136; p = .001*;  95% CI DV = [1.35 ; 5.95]; Cohen's d = 0.98 | 4.57 (4.71) | 5.05 (4.13) | t(68) = -0.456; p = .650;  95% CI DV = [-2.6 ; 1.63]; Cohen's d = 0.11 |
| **Phoneme deletion TP2**  **(items solved)** | 10.4 (3.62) | 5.33 (4.88) | U = 128; p = .001*;  95% CI DV = [2.64 ; 7.49]; Cohen's d = 1.29 | 9.67 (3.19) | 10.40 (3.62) | t(68) = -0.882; p = .381;  95% CI DV = [-2.39 ; 0.93]; Cohen's d = 0.22 |
| **Phoneme deletion TP3**  **(items solved)** | 13.63 (4.74) | 8.40 (5.19) | U = 131; p = .001*;  95% CI DV = [2.27 ; 8.18]; Cohen's d = 1.09 | 13.45 (4.49) | 13.63 (4.74) | t(67) = -0.156; p = .876;  95% CI DV = [-2.43 ; 2.08]; Cohen's d = 0.04 |
| **Rapid naming colours & objects TP1 (seconds)** | 122.7 (28.79) | 144.27 (31.76) | U = 167; p = .012;  95% CI DV = [-39.55 ; -3.59]; Cohen's d = 0.74 | 129.73 (26.24) | 122.70 (28.79) | t(68) = 1.05; p = .297;  95% CI DV = [-6.33 ; 20.4]; Cohen's d = 0.26 |
| **Rapid naming colours & objects TP2 (seconds)** | 30.13 (7.42) | 42.36 (17.43) | U = 138; p = .006;  95% CI DV = [-19.04 ; -5.42]; Cohen's d = 1.15 | 29.9 (7.52) | 30.13 (7.42) | t(67) = -0.126; p = .900;  95% CI DV = [-3.85 ; 3.39]; Cohen's d = 0.03 |
| **Rapid naming colours & objects TP3 (seconds)** | 106.13 (20.82) | 130.73 (36.96) | U = 160; p = .008;  95% CI DV = [-40.44 ; -8.78]; Cohen's d = 0.96 | 109.13 (20.03) | 106.13 (20.82) | t(68) = 0.608; p = .545;  95% CI DV = [-6.87 ; 12.88]; Cohen's d = 0.15 |
| **Rapid naming letters & digits TP2 (seconds)** | 93.38 (15.86) | 114.53 (24.46) | U = 128; p = .001*;  95% CI DV = [-32.41 ; -9.91]; Cohen's d = 1.16 | 94.79 (17.61) | 93.38 (15.86) | t(67) = 0.35; p = .727;  95% CI DV = [-6.67 ; 9.51]; Cohen's d = 0.09 |
| **Rapid naming letters & digits TP3 (seconds)** | 52.5 (9.81) | 65.93 (19.05) | U = 150; p = .005;  95% CI DV = [-21.28 ; -5.59]; Cohen's d = 1.06 | 50.90 (8.71) | 52.50 (9.81) | t(67) = -0.702; p = .485;  95% CI DV = [-6.16 ; 2.96]; Cohen's d = 0.17 |

* Remains significant after Bonferroni correction for multiple comparisons, i.e. six to ten comparisons at each TP.

Table S3. Tests diagnosing dyslexia: children with familial history of dyslexia and typical readers (TR: typical readers, DR: children with dyslexia, FHD-: children without familial history of dyslexia, FHD+: children with familial history of dyslexia).

|  | **FHD+** | |  | **TR** | |  |
| --- | --- | --- | --- | --- | --- | --- |
|  | **TR** | **DR** |  | **FHD-** | **FHD+** |  |
|  | n = 40 | n = 15 |  | n = 30 | n = 40 |  |
| **Word reading** | 6.68 (1.72) | 3.40 (1.40) | U = 30; p < .001*;  95% CI DV = [2.28 ; 4.27]; Cohen's d = 2.04 | 6.38 (1.94) | 6.68 (1.72) | t(67) = -0.67; p = .505;  95% CI DV = [-1.18 ; 0.59]; Cohen's d = 0.17 |
| **Pseudo-word reading** | 5.63 (1.51) | 2.87 (1.19) | U = 46; p < .001*;  95% CI DV = [1.89 ; 3.63]; Cohen's d = 1.96 | 5.62 (1.37) | 5.63 (1.51) | t(67) = -0.012; p = .990;  95% CI DV = [-0.71 ; 0.71]; Cohen's d = 0.01 |
| **Reading with lexical decision** | 6.03 (1.64) | 2.6 (0.99) | U = 11; p < .001*;  95% CI DV = [2.52 ; 4.33]; Cohen's d = 2.34 | 6.38 (1.59) | 6.03 (1.64) | t(67) = 0.897; p = .373;  95% CI DV = [-0.43 ; 1.14]; Cohen's d = 0.22 |
| **Text reading** | 5.48 (1.95) | 2.47 (1.06) | U = 53; p < .001*;  95% CI DV = [1.94 ; 4.08]; Cohen's d = 1.74 | 5.93 (2.21) | 5.48 (1.95) | t(66) = 0.894; p = .375;  95% CI DV = [-0.56 ; 1.47]; Cohen's d = 0.22 |
| **Text writing** | 4.98 (1.89) | 1.93 (1.34) | U = 63; p < .001*;  95% CI DV = [1.97 ; 4.11]; Cohen's d = 1.77 | 4.90 (2.21) | 4.98 (1.89) | t(67) = -0.159; p = .874;  95% CI DV = [-1.07 ; 0.91]; Cohen's d = 0.04 |
| **Word writing** | 4.43 (1.75) | 2.00 (1.41) | U = 92; p < .001*;  95% CI DV = [1.41 ; 3.44]; Cohen's d = 1.48 | 4.55 (2.29) | 4.43 (1.75) | t(67) = 0.26; p = .795;  95% CI DV = [-0.85 ; 1.1]; Cohen's d = 0.06 |
| **Phoneme deletion** | 5.20 (1.92) | 3.40 (2.20) | U = 161; p = .007;  95% CI DV = [0.59 ; 3.02]; Cohen's d = 0.92 | 5.55 (1.79) | 5.20 (1.92) | t(67) = 0.772; p = .443;  95% CI DV = [-0.56 ; 1.26]; Cohen's d = 0.19 |
| **Battery of phonological tasks** | 5.05 (2.36) | 3.47 (1.73) | U = 169; p = .012;  95% CI DV = [0.24 ; 2.93]; Cohen's d = 0.73 | 5.38 (1.45) | 5.05 (2.36) | t(67) = 0.664; p = .509;  95% CI DV = [-0.66 ; 1.32]; Cohen's d = 0.16 |
| **Pseudo-word repetition** | 5.35 (1.79) | 3.60 (1.99) | U = 152; p = .005;  95% CI DV = [0.63 ; 2.87]; Cohen's d = 0.97 | 4.97 (1.50) | 5.35 (1.79) | t(67) = -0.941; p = .350;  95% CI DV = [-1.2 ; 0.43]; Cohen's d = 0.23 |

* Remains significant after Bonferroni correction for nine comparisons. Note: sten scores are reported (population M = 5.5, SD = 2.0).

Table S4. fMRI experiment: children with familial history of dyslexia and typical readers (TR: typical readers, DR: children with dyslexia, FHD-: children without familial history of dyslexia, FHD+: children with familial history of dyslexia).

|  | **FHD+** | |  | **TR** |  |  |
| --- | --- | --- | --- | --- | --- | --- |
|  | **TR** | **DR** |  | **FHD-** | **FHD+** |  |
|  | n = 40 | n = 15 |  | n = 30 | n = 40 |  |
| **Rhyme task: accuracy TP1**  **(percent of correct responses)** | 90.50 (15.22) | 75.33 (23.94) | U = 148; p = .003*;  95% CI DV = [4.27 ; 26.06]; Cohen's d = 0.86 | 94.66 (8.34) | 90.5 (15.22) | t(67) = 1.33; p = .188;  95% CI DV = [-2.08 ; 10.39]; Cohen's d = 0.33 |
| **Rhyme task: accuracy TP3**  **(percent of correct responses)** | 71.50 (21.16) | 62.14 (19.68) | U = 199; p = .109;  95% CI DV = [-3.6 ; 22.32]; Cohen's d = 0.46 | 72.07 (19.23) | 71.5 (21.16) | t(66) = 0.114; p = .910;  95% CI DV = [-9.46 ; 10.6]; Cohen's d = 0.03 |
| **Voice task: accuracy TP1**  **(percent of correct responses)** | 95.26 (6.17) | 91.33 (9.90) | U = 228; p = .189;  95% CI DV = [-0.57 ; 8.41]; Cohen's d = 0.54 | 92.17 (15.35) | 95.26 (6.17) | t(67) = -1.144; p = .257;  95% CI DV = [-8.48 ; 2.3]; Cohen's d = 0.28 |
| **Voice task: accuracy TP3**  **(percent of correct responses)** | 86.54 (14.96) | 83.33 (16.11) | U = 264; p = .570;  95% CI DV = [-6.11 ; 12.52]; Cohen's d = 0.21 | 89.33 (11.58) | 86.54 (14.96) | t(67) = 0.846; p = .400;  95% CI DV = [-3.8 ; 9.39]; Cohen's d = 0.21 |
| **Rhyme task: reaction times TP1**  **(seconds)** | 1.79 (0.50) | 1.92 (0.37) | U = 255; p = .395;  95% CI DV = [-0.42 ; 0.16]; Cohen's d = 0.28 | 1.59 (0.34) | 1.79 (0.50) | t(67) = -1.829; p = .072;  95% CI DV = [-0.41 ; 0.02]; Cohen's d = 0.45 |
| **Rhyme task: reaction times TP3**  **(seconds)** | 2.09 (0.56) | 2.17 (0.57) | U = 273; p = .890;  95% CI DV = [-0.43 ; 0.27]; Cohen's d = 0.14 | 1.97 (0.40) | 2.09 (0.56) | t(66) = -0.991; p = .325;  95% CI DV = [-0.37 ; 0.12]; Cohen's d = 0.25 |
| **Voice task: reaction times TP1**  **(seconds)** | 2.06 (0.58) | 1.91 (0.55) | U = 227; p = .202;  95% CI DV = [-0.2 ; 0.5]; Cohen's d = 0.27 | 1.84 (0.62) | 2.06 (0.58) | t(67) = -1.484; p = .142;  95% CI DV = [-0.5 ; 0.07]; Cohen's d = 0.37 |
| **Voice task: reaction times TP3**  **(seconds)** | 2.21 (0.54) | 2.19 (0.47) | U = 282; p = .839;  95% CI DV = [-0.3 ; 0.33]; Cohen's d = 0.03 | 2.03 (0.51) | 2.21 (0.54) | t(67) = -1.404; p = .165;  95% CI DV = [-0.43 ; 0.08]; Cohen's d = 0.35 |
| **Rhyme task: rejected volumes TP1** | 1.98 (3.25) | 3.8 (3.32) | U = 180; p = .016;  95% CI DV = [-3.81 ; 0.16]; Cohen's d = 0.57 | 3.80 (4.55) | 1.98 (3.25) | t(68) = 1.959; p = .054;  95% CI DV = [-0.03 ; 3.68]; Cohen's d = 0.48 |
| **Rhyme task: rejected volumes TP3** | 2.78 (4.16) | 5.73 (4.62) | U = 175; p = .013;  95% CI DV = [-5.56 ; -0.36]; Cohen's d = 0.70 | 2.47 (3.78) | 2.78 (4.16) | t(68) = -0.319; p = .751;  95% CI DV = [-2.24 ; 1.62]; Cohen's d = 0.08 |
| **Voice task: rejected volumes TP1** | 2.93 (4.78) | 2.8 (3.47) | U = 280; p = .682;  95% CI DV = [-2.59 ; 2.84]; Cohen's d = 0.03 | 3.77 (5.75) | 2.93 (4.78) | t(68) = 0.668; p = .506;  95% CI DV = [-1.67 ; 3.36]; Cohen's d = 0.16 |
| **Voice task: rejected volumes TP3** | 3.15 (5.14) | 5.2 (5.66) | U = 211; p = .067;  95% CI DV = [-5.26 ; 1.16]; Cohen's d = 0.40 | 2.80 (4.49) | 3.15 (5.14) | t(68) = -0.297; p = .767;  95% CI DV = [-2.7 ; 2]; Cohen's d = 0.07 |

Table S5. Significant activation to Rhyme>Voice contrast in TR and DR children

|  | **Brain region** | **H** | | **x** | **y** | | **z** | | | **t** | | **p** | | **Voxels** |
| --- | --- | --- | --- | --- | --- | --- | --- | --- | --- | --- | --- | --- | --- | --- |
| **TR** | **TP1** |  | |  |  | |  | | |  | |  | |  |
|  | Lingual (L, R), Putamen (L), Inferior Frontal (orb&tri, L), Hippocampus (L, R), Fusiform (L), Calcarine (L,R), Putamen (R), Caudate (R), Amygdala (L, R), Inferior temporal (R ),Middle Temporal Gyrus (R) | L, R | | -26 | 2 | | -16 | | | 5.35 | | <.001 | | 9810 |
|  | Middle & Superior Occipital, Cuneus | L | | -32 | -82 | | 22 | | | 3.93 | | <.001 | | 1062 |
|  | Medial & Superior Frontal (L), Anterior Cingulate (L,R) | L,R | | -6 | 58 | | 20 | | | 3.72 | | <.001 | | 529 |
|  | Superior Frontal | R | | 14 | 56 | | 26 | | | 3.66 | | <.001 | | 126 |
|  | Middle Temporal & Occipital, Inferior Temporal & Occipital | L | | -52 | -66 | | 2 | | | 3.65 | | <.001 | | 394 |
|  | Precentral | L | | -54 | -4 | | 48 | | | 3.55 | | <.001 | | 57 |
|  | Middle Temporal & Occipital, Superior Temporal | R | | 44 | -80 | | 14 | | | 3.46 | | <.001 | | 596 |
|  | Superior & Inferior Parietal | L | | -24 | -58 | | 54 | | | 3.44 | | <.001 | | 186 |
|  | Middle Cingulum | L,R | | 0 | 4 | | 36 | | | 3.42 | | .001 | | 118 |
|  | Calcarine | L | | -8 | -86 | | 2 | | | 3.39 | | .001 | | 66 |
|  | Superior Occipital | R | | 22 | -74 | | 40 | | | 3.20 | | .001 | | 85 |
|  | Caudate | L | | -14 | 26 | | 4 | | | 3.19 | | .001 | | 115 |
|  | Superior Parietal | R | | 28 | -56 | | 66 | | | 3.09 | | .001 | | 65 |
|  | Cuneus | R | | 12 | -88 | | 26 | | | 2.98 | | .002 | | 54 |
|  | Medial & Superior Frontal | L | | -22 | 52 | | 32 | | | 2.90 | | .002 | | 82 |
|  |  |  | |  |  | |  | | |  | |  | |  |
|  | **TP3** |  | |  |  | |  | | |  | |  | |  |
|  | Middle & Superior Occipital | R | | 42 | -84 | | 22 | | | 4.85 | | <.001 | | 263 |
|  | Putamen, Caudate | L | | -16 | 12 | | -4 | | | 4.09 | | <.001 | | 524 |
|  | Putamen, Caudate | R | | 16 | 12 | | -4 | | | 3.99 | | <.001 | | 548 |
|  | Middle & Superior Occipital | L | | -34 | -90 | | 24 | | | 3.66 | | <.001 | | 295 |
|  | Parahippocampal, Fusiform | R | | 24 | -38 | | -10 | | | 3.58 | | <.001 | | 166 |
| **TR FHD+** | **TP1** |  | |  |  | |  | | |  | |  | |  |
|  | Fusiform | L | | -22 | -42 | | -14 | | | 3.52 | | <.001 | | 84 |
|  |  |  | |  |  | |  | | |  | |  | |  |
|  | **TP3** |  | |  |  | |  | | |  | |  | |  |
|  | Postcentral Gyrus | L | | -46 | -24 | | 60 | | | 3.42 | | <.001 | | 56 |
| **DR** | **TP1** |  | |  |  | |  | | |  | |  | |  |
|  | Insula, Precentral & Postcentral Gyri | R | | 38 | -10 | | 12 | | | 3.32 | | .002 | | 139 |
|  |  |  | |  |  | |  | | |  | |  | |  |
|  | **TP3** |  | |  |  | |  | | |  | |  | |  |
|  | Superior Temporal, Precentral, Rolandic Operculum, Heschl, Postcentral, Temporal Pole, Middle Temporal, Supp Moto Area, Putamen | R | | 30 | -30 | | 20 | | | 5.36 | | <.001 | | 3597 |
|  | Middle & Inferior Temporal |  | | -58 | -52 | | -4 | | | 5.29 | | <.001 | | 367 |
|  | Superior & Middle Temporal, Heschl | L | | -52 | -26 | | 6 | | | 5.20 | | <.001 | | 577 |
|  | Superior Temporal, Rolandic Oper | L | | -50 | 6 | | -12 | | | 4.96 | | <.001 | | 445 |
|  | Insula | L | | -28 | -30 | | 20 | | | 4.82 | | <.001 | | 90 |
|  | Middle Occipital | L | | -40 | -86 | | 22 | | | 4.80 | | <.001 | | 189 |
|  | Inferior Frontal (orb, tri) | L | | -48 | 32 | | 4 | | | 4.78 | | <.001 | | 394 |
|  | Precentral & Postcentral, Inferior Frontal (oper) |  | | -60 | 2 | | 26 | | | 4.72 | | <.001 | | 765 |
|  | Putamen, Inferior Frontal (orb), Temporal Pole (sup), Caudate, Amygdala | L | | -26 | 6 | | -24 | | | 4.57 | | <.001 | | 1412 |
|  | Putamen, Amygdala, Hippocampus, Temporal Pole (sup) | R | | 10 | -30 | | -10 | | | 4.55 | | <.001 | | 803 |
|  | Precentral & Postcentral, Inferior Frontal (oper) | R | | 30 | -30 | | 74 | | | 4.28 | | <.001 | | 171 |
|  | Thalamus | R | | 12 | -20 | | 6 | | | 4.19 | | <.001 | | 127 |
|  | Cerebellum (IV, V), ParaHippocampal, Fusiform | L | | -6 | -48 | | -8 | | | 4.15 | | <.001 | | 208 |
|  | Thalamus | L | | -10 | -20 | | 2 | | | 4.06 | | <.001 | | 85 |
|  | Middle Cingulate | L | | -2 | -16 | | 46 | | | 3.18 | | .002 | | 77 |
| **DR FHD+** | **TP1** |  | |  |  | |  | | |  | |  | |  |
|  | -  **TP3** |  | |  |  | |  | | |  | |  | |  |
|  | Middle Cingulate | R | | 18 | -10 | | 46 | | | 6.41 | | <.001 | | 515 |
|  | Hippocampus, Amygdala, Parahippocampal | R | | 24 | -14 | | -14 | | | 5.45 | | <.001 | | 747 |
|  | Middle Occipital | L | | -44 | -86 | | 20 | | | 5.19 | | <.001 | | 86 |
|  | Superior & Middle Temporal, Heschl | L | | -52 | -26 | | 4 | | | 5.13 | | <.001 | | 284 |
|  | Inferior Frontal (orb), Superior Temporal, Parahippocampal | L | | -30 | 26 | | -22 | | | 4.94 | | <.001 | | 218 |
|  | Supramarginal | L | | -50 | -42 | | 28 | | | 4.80 | | <.001 | | 105 |
|  | Precentral & Postcentral | L | | -64 | 8 | | 22 | | | 4.73 | | <.001 | | 160 |
|  | Rolandic Operculum, Insula, Heschl , Superior Temporal | R | | 30 | -6 | | 24 | | | 4.72 | | <.001 | | 559 |
|  | Inferior Frontal Gyrus (tri, orb) | L | | -50 | 34 | | 8 | | | 4.42 | | <.001 | | 152 |
|  | Superior & Middle Temporal | R | | 50 | 2 | | -10 | | | 4.25 | | <.001 | | 182 |
|  | Lingual | R | | 10 | -30 | | -10 | | | 4.24 | | <.001 | | 71 |
|  | Superior & Middle Temporal | R | | 52 | -34 | | 4 | | | 4.22 | | <.001 | | 367 |
|  | Insula | L | | -28 | -30 | | 18 | | | 4.16 | | <.001 | | 56 |
|  | Insula, Putamen | L | | -26 | 12 | | 14 | | | 3.98 | | .001 | | 51 |
|  | Middle Temporal | L | | -58 | -52 | | -2 | | | 3.97 | | .001 | | 72 |
|  | Cingulate (mid) | L | | -14 | -14 | | 48 | | | 3.80 | | .001 | | 56 |
|  | Precentral | R | | 38 | -14 | | 44 | | | 3.76 | | .001 | | 136 |
|  |  |  |  | | |  | |  |  | |  | |  | |

Table S6. Significant activation to Rhyme>Voice contrast in FHD- and FHD+ children

|  | **Brain region** | **H** | **x** | **y** | **z** | **t** | **p** | **Voxels** |
| --- | --- | --- | --- | --- | --- | --- | --- | --- |
| **FHD-** | **TP1** |  |  |  |  |  |  |  |
|  | Insula (L, R), Inferior Frontal (orb, oper, tri, L, R), Anterior Cingulate (L, R), Putamen (L, R), Precentral (L), Superior Temporal (L, R), Lingual  (L, R), Medial Frontal (sup, orb, L), Hippocamus (L, R), Fusiform (L, R), Postcentral (L), Caudate (L, R), Middle Temporal (L, R), Calcarine (L, R), Middle Cingulate (L, R), Cerebellum (III, IV, V, L, R), Parahippocampal (L), Middle Occipital (R), Rolandic Operculum (L), Inferior Temporal (R), Amygdala (L, R), Pallidum (L), Precuneus (L, R), Thalamus (L, R), Heschl (L) | L,R | -24 | 0 | -16 | 6.29 | <.001 | 28464 |
|  | Superior Parietal | R | 26 | -56 | 72 | 3.77 | <.001 | 56 |
|  | Superior Temporal, Rolandic Operculum, Postcentral | R | 70 | -10 | 22 | 3.68 | <.001 | 516 |
|  | Superior Parietal | L | -26 | -54 | 56 | 3.50 | .001 | 221 |
|  | Middle & Superior Occipital | L | -38 | -78 | 20 | 3.42 | .001 | 261 |
|  | Supplementary Motor Area | L | -6 | 4 | 64 | 3.42 | .001 | 58 |
|  | Cuneus | R | 14 | -90 | 28 | 3.16 | .002 | 51 |
|  | Superior Frontal | L | -16 | 26 | 44 | 3.10 | .002 | 51 |
|  |  |  |  |  |  |  |  |  |
|  | **TP3** |  |  |  |  |  |  |  |
|  | Middle & Superior Occipital | R | 40 | -86 | 22 | 5.41 | <.001 | 550 |
|  | Putamen (L, R), Caudate (L, R), Pallidum (L, R), Insula (L, R), Amygdala (L), Hippocampus (L), Inferior Frontal (orb, L) | L, R | -6 | 18 | -4 | 5.01 | <.001 | 2600 |
|  | Middle & Inferior Temporal, Lingual, Middle & Inferior Occipital, Fusiform, Parahippocampal | L | -30 | -46 | -4 | 4.35 | <.001 | 1141 |
|  | Supramarginal | R | 62 | -24 | 50 | 4.34 | <.001 | 73 |
|  | Middle & Superior Occipital | L | -32 | -84 | 16 | 4.23 | <.001 | 788 |
|  | Precentral | R | 38 | -22 | 70 | 4.05 | <.001 | 112 |
|  | Parahippocampal, Fusiform, Precuneus, Calcarine | R | 28 | -46 | 0 | 4.03 | <.001 | 390 |
|  | Medial Frontal (sup) | L | -4 | 52 | 18 | 3.83 | <.001 | 152 |
|  | Precentral | R | 58 | 4 | 32 | 3.77 | <.001 | 69 |
|  | Inferior Occipital | R | 36 | -64 | -8 | 3.77 | <.001 | 52 |
|  | Thalamus | L | -6 | -30 | 2 | 3.75 | <.001 | 99 |
|  | Anterior Cingulate | L | -6 | 54 | 2 | 3.50 | .001 | 221 |
|  | Superior temporal, Temporal Pole (sup) | R | 36 | 8 | -18 | 3.40 | .001 | 68 |
| **FHD- TR** | **TP1** |  |  |  |  |  |  |  |
|  | Insula (L, R), Medial Frontal (sup, orb, L, R), Anterior Cingulate (L, R), Putamen (L, R), Inferior  Frontal (orb, oper, tri, L), Lingual (L), Superior Temporal, (L, R), Precentral (L), Caudate (L, R), Hippocamus (L, R), Middle Temporal (L), Fusiform (L), Postcentral (L), Parahippocampal (L), Middle Cingulate (L, R), Cerebellum (IV, V, L), Amygdala (L), Pallidum (L,R), Precuneus (L), Rolandic Operculum (L), Thalamus(L), Heschl (L) | L,R | -24 | 0 | -18 | 5.66 | <.001 | 19562 |
|  | Lingua, Inferior, Middle & Superior Temporal, Fusiform , Calcarine, Parahippocampal , Precuneus | R | 30 | -54 | -2 | 4.97 | <.001 | 3337 |
|  | Precentral | R | 40 | -26 | 70 | 4.84 | <.001 | 61 |
|  | Superior Parietal Lobule | R | 34 | -52 | 70 | 4.10 | <.001 | 72 |
|  | Superior Temporal, Rolandic Operculum | L | -40 | -38 | 16 | 3.65 | .001 | 141 |
|  | Postcentral | R | 70 | -8 | 24 | 3.53 | .001 | 56 |
|  | Superior & Inferior Parietal Lobule | L | -26 | -54 | 56 | 3.40 | .001 | 293 |
|  | Superior & Middle Occipital | L | -18 | -88 | 34 | 3.26 | .001 | 73 |
|  |  |  |  |  |  |  |  |  |
|  | **TP3** |  |  |  |  |  |  |  |
|  | Superior & Middle Occipital | R | 38 | -88 | 22 | 5.34 | <.001 | 341 |
|  | Putamen (R,L), Caudate (R,L), Pallidium (R,L) | R,L | -8 | 20 | -2 | 4.74 | <.001 | 1201 |
|  | Medial Frontal | L | -6 | 52 | 18 | 3.83 | <.001 | 144 |
|  | Putamen | L | -24 | -6 | 22 | 3.59 | .001 | 82 |
|  | Superior & Middle Occipital | L | -32 | -84 | 16 | 3.58 | .001 | 246 |
|  | Fusiform, Parahippocampal | L,R | 28 | -46 | 0 | 3.47 | .001 | 134 |
|  | Middle Temporal | L | -56 | -62 | 4 | 3.18 | .002 | 59 |
|  | Middle Occipital | L | -40 | -56 | 2 | 3.13 | .002 | 96 |
| **FHD+** | **TP1** |  |  |  |  |  |  |  |
|  | Parahippocampal | L | -28 | -32 | -18 | 3.60 | <.001 | 120 |
|  | Vermis (IV, V) | L | 2 | -46 | 2 | 3.16 | .001 | 56 |
|  | Putamen | L | -12 | 10 | -8 | 3.11 | .001 | 80 |
|  |  |  |  |  |  |  |  |  |
|  | **TP3** |  |  |  |  |  |  |  |
|  | Middle Cingulate | R | 18 | -8 | 40 | 5.13 | <.001 | 255 |
|  | Middle Occipital | L | -38 | -88 | 24 | 3.95 | <.001 | 109 |
|  | Inferior & Middle Frontal (orb) | L | -38 | 40 | -8 | 3.40 | .001 | 91 |
|  | Putamen | R | 18 | 8 | -6 | 3.36 | .001 | 88 |
|  | Putamen | L | -18 | 14 | -10 | 3.15 | .001 | 61 |
| **FHD+ TR** | **TP1** |  |  |  |  |  |  |  |
|  | Fusiform | L | -22 | -42 | -14 | 3.52 | <.001 | 84 |
|  |  |  |  |  |  |  |  |  |
|  | **TP3** |  |  |  |  |  |  |  |
|  | Postcentral Gyrus | L | -46 | -24 | 60 | 3.42 | <.001 | 56 |

Table S7. Significant group and time point effects across groups of children with (DR) and without dyslexia (TR) restricted to FHD+ children

|  | **Brain region** | **H** | **x** | **y** | **z** | **t** | **p** | **Voxels** |
| --- | --- | --- | --- | --- | --- | --- | --- | --- |
| **Group Effects** | **TP1 TR > DR** |  |  |  |  |  |  |  |
|  | - |  |  |  |  |  |  |  |
|  | **TP1 DR > TR** |  |  |  |  |  |  |  |
|  | Middle & Superior Frontal Gyri | R | 34 | 26 | 56 | 4.01 | <.001 | 170 |
|  | Inferior Parietal Lobule | R | 52 | -50 | 58 | 3.92 | <.001 | 84 |
|  | Middle & Superior Frontal Gyri | R | 38 | 0 | 64 | 3.56 | <.001 | 92 |
|  | Middle Frontal & Precentral Gyri | L | -50 | 14 | 48 | 3.42 | .001 | 135 |
|  | **TP3 TR > DR** |  |  |  |  |  |  |  |
|  | - |  |  |  |  |  |  |  |
|  | **TP3 DR > TR** |  |  |  |  |  |  |  |
|  | Superior Temporal & Supramarginal Gyri | L | -48 | -44 | 26 | 5.59 | <.001 | 241 |
|  | Superior & Middle Temporal Gyrus, Hippocampus, Putamen, Supramarginal Gyrus, Amygdala, Thalamus, Caudate, ParaHippocampal, Heschl Gyrus, Inferior Temporal Gyrus, Inferior Frontal Gyrus (orb), Fusiform | R | 32 | -32 | 24 | 5.15 | <.001 | 5732 |
|  | Precentral & Postcentral Gyri | L | -54 | 0 | 24 | 4.15 | <.001 | 318 |
|  | Superior Temporal Gyrus, Thalamus, Caudate, Heschl Gyrus, Rolandic Operculum | L | -26 | -30 | 16 | 4.08 | <.001 | 1578 |
|  | Putamen | L | -20 | -4 | -6 | 3.54 | <.001 | 197 |
|  | Caudate | R | 14 | 10 | 22 | 3.50 | <.001 | 54 |
|  | Middle Temporal Gyrus | L | -52 | -48 | 6 | 3.48 | .001 | 149 |
|  | Angular Gyrus | R | 52 | -66 | 30 | 3.48 | .001 | 89 |
|  | Middle Frontal Gyrus | L | -26 | 18 | 44 | 3.40 | .001 | 62 |
|  | Superior Temporal Gyrus | L | -30 | 6 | -24 | 3.25 | .001 | 84 |
|  | Superior Frontal Gyrus | R | 26 | 36 | 52 | 3.22 | .002 | 50 |
|  | Middle Cingulum | L,R | 4 | -32 | 50 | 2.99 | .002 | 113 |
| **TP Effects** | **TR TP3 > TP1** |  |  |  |  |  |  |  |
|  | Precentral Gyrus | L | -42 | -26 | 66 | 3.75 | <.001 | 213 |
|  |  |  |  |  |  |  |  |  |
|  | **DR TP3 > TP1** |  |  |  |  |  |  |  |
|  | Superior & Middle Temporal Gyrus, Insula | R | 38 | 20 | -32 | 3.81 | <.001 | 127 |
| **Interaction** |  |  |  |  |  |  |  |  |
|  | - |  |  |  |  |  |  |  |

Note: Group effects were tested with one-way ANOVA, TP effects were tested with paired t-tests, Group and TP interaction was tested with flexible factorial design.

Table S8. Significant group and time point effects across groups of children with (FHD+) and without familial history of dyslexia (FHD-) restricted to typical readers (TR).

|  | **Brain region** | **H** | **x** | **y** | **z** | **t** | **p** | **Voxels** |
| --- | --- | --- | --- | --- | --- | --- | --- | --- |
| **Group Effects** | **TP1 FHD- > FHD+** |  |  |  |  |  |  |  |
|  | Superior & Middle Temporal Gyri | L | -46 | -28 | -2 | 4.31 | <.001 | 1203 |
|  | Superior& Middle Temporal Gyri, Inferior Temporal Gyrus (orb), Insula, Putamen | L | -42 | 16 | -16 | 4.25 | <.001 | 1153 |
|  | Thalamus | L | -4 | -4 | 2 | 3.78 | <.001 | 78 |
|  | Superior Temporal Gyrus, Insula | R | 44 | 4 | -18 | 3.74 | <.001 | 241 |
|  | Superior Temporal Gyrus, Rolandic Operculum | L | -58 | -10 | 4 | 3.57 | <.001 | 100 |
|  | Precentral Gyrus, Middle Frontal Gyrus, Inferior Frontal Gyrus (oper, tri) | L | -42 | -2 | 46 | 3.56 | <.001 | 592 |
|  | Postcentral Gyrus | L | -42 | -32 | 66 | 3.46 | <.001 | 77 |
|  | Superior & Medial Frontal Gyri | L | 0 | 50 | 40 | 3.43 | .001 | 293 |
|  | Middle Temporal Gyrus | R | 52 | -74 | 10 | 3.33 | .001 | 78 |
|  | Precuneus | L | -24 | -48 | 6 | 3.21 | .001 | 67 |
|  | Postcentral & Precentral Gyri, Paracentral Lobule | L | -18 | -18 | 70 | 3.16 | .001 | 329 |
|  | Superior & Medial Frontal Gyri | L | -24 | 62 | -2 | 3.12 | .001 | 124 |
|  | Lingual | R | 12 | -30 | -10 | 3.11 | .001 | 50 |
|  | Postcentral | R | 70 | -12 | 24 | 3.07 | .002 | 50 |
|  | Inferior Parietal Lobule, Postcentral | L | -24 | -48 | 54 | 3.06 | .002 | 76 |
|  | Superior & Middle Temporal Gyri | R | 38 | -38 | 4 | 3.06 | .002 | 196 |
|  | Superior & Medial Frontal Gyri | R | 6 | 58 | -2 | 3.04 | .002 | 108 |
|  | **TP1 FHD+ > FHD-** |  |  |  |  |  |  |  |
|  | - |  |  |  |  |  |  |  |
|  | **TP3 FHD- > FHD+** |  |  |  |  |  |  |  |
|  | Caudate, Anterior Cingulate | L | -6 | 16 | -6 | 3.84 | <.001 | 197 |
|  | Caudate | L | -26 | -8 | 18 | 3.76 | <.001 | 209 |
|  | Putamen | L | -20 | -4 | -8 | 3.59 | <.001 | 186 |
|  | Superior & Middle Temporal Gyrus | R | 38 | 4 | -22 | 3.58 | <.001 | 123 |
|  | Middle & Superior Teporal Gyrus | R | 46 | -6 | -14 | 3.37 | .001 | 134 |
|  | Superior Frontal Gyrus | L | -6 | 52 | 20 | 3.35 | .001 | 79 |
|  | Middle & Superior Occipital Gyri | L | 34 | -90 | 24 | 3.35 | .001 | 58 |
|  | Medial Frontal Gyrus (orb) | R | 4 | 54 | -10 | 3.10 | .001 | 55 |
|  | **TP3 FHD+ > FHD-** |  |  |  |  |  |  |  |
|  | - |  |  |  |  |  |  |  |
| **TP Effects** | **FHD- TP1 > TP3** |  |  |  |  |  |  |  |
|  | Superior Temporal Gyrus, Rolandic Operculum, Heshl Gyrus, Insula, Postcentral Gyrus | L | -38 | -10 | 2 | 3.79 | <.001 | 607 |
|  | Inferior Frontal Gyrus (oper&tri) | L | -50 | 12 | 22 | 3.60 | .001 | 170 |
|  | Hippocampus | R | 34 | -32 | 4 | 3.60 | .001 | 82 |
|  | Superior Temporal Gyrus, Rolandic Operculum | L | -42 | -42 | 22 | 3.44 | .001 | 99 |
|  | Inferior Parietal Lobule | L | -36 | -44 | 42 | 3.79 | .001 | 50 |
|  |  |  |  |  |  |  |  |  |
|  | **FHD+ TP3 > TP1** |  |  |  |  |  |  |  |
|  | Precentral Gyrus | L | -42 | -26 | 66 | 3.75 | <.001 | 213 |
| **Interaction** | **FHD- TP1>TP3 & FHD+ TP3>TP1** |  |  |  |  |  |  |  |
|  | Postcentral & Precentral Gyri | L | -46 | -24 | 60 | 3.36 | <.001 | 223 |

Note: Group effects were tested with one-way ANOVA, TP effects were tested with paired t-tests, Group and TP interaction was tested with flexible factorial design.
